# Better to be in agreement than in bad company: A critical analysis of many kappa-like tests

**DOI:** 10.3758/s13428-022-01950-0

**Published:** 2022-09-16

**Authors:** Paulo Sergio Panse Silveira, Jose Oliveira Siqueira

**Affiliations:** 1 Department of Pathology (LIM01-HCFMUSP), Medical School, University of Sao Paulo, Av. Dr. Arnaldo 455, room 1103, Sao Paulo, SP 01246-903 Brazil; 2 Department of Legal Medicine, Bioethics, Occupational Medicine and Physical Medicine and Rehabilitation, Medical School, University of Sao Paulo, Av. Dr. Arnaldo 455, room 1103, Sao Paulo, 01246-903 Brazil

**Keywords:** Agreement coefficient, Contingency table, Categorical data analysis, Inter-rater reliability, 62H17, 62H20, 62P10, 62P15, 92-04, 92B15

## Abstract

We assessed several agreement coefficients applied in 2x2 contingency tables, which are commonly applied in research due to dichotomization. Here, we not only studied some specific estimators but also developed a general method for the study of any estimator candidate to be an agreement measurement. This method was developed in open-source R codes and it is available to the researchers. We tested this method by verifying the performance of several traditional estimators over all possible configurations with sizes ranging from 1 to 68 (total of 1,028,789 tables). Cohen’s kappa showed handicapped behavior similar to Pearson’s r, Yule’s Q, and Yule’s Y. Scott’s pi, and Shankar and Bangdiwala’s B seem to better assess situations of disagreement than agreement between raters. Krippendorff’s alpha emulates, without any advantage, Scott’s pi in cases with nominal variables and two raters. Dice’s F1 and McNemar’s chi-squared incompletely assess the information of the contingency table, showing the poorest performance among all. We concluded that Cohen’s kappa is a measurement of association and McNemar’s chi-squared assess neither association nor agreement; the only two authentic agreement estimators are Holley and Guilford’s G and Gwet’s AC1. The latter two estimators also showed the best performance over the range of table sizes and should be considered as the first choices for agreement measurement in contingency 2x2 tables. All procedures and data were implemented in R and are available to download from Harvard Dataverse https://doi.org/10.7910/DVN/HMYTCK.

**Supplementary Information:**

The online version contains supplementary material available at 10.3758/s13428-022-01950-0.

## Introduction

Contingency tables are prevalent and, among them, 2x2 tables reign. Conclusions drawn from them depend on statistical measures. In psychology, many observations are somewhat fuzzy and thus careful researchers must verify whether different assessors agree, which is usually an application of Cohen’s *κ* test (Banerjee, Capozzoli, McSweeney, & Sinha, 1999; Gwet, 2008; Gwet, 2010). In healthcare, clinical trials depend on the application of drugs, devices, or procedures for checking their association with potentially positive or negative effects, which makes Fisher’s exact and chi-squared tests largely used (Ludbrook, 2011). In epidemiology, researchers also need to assess agreement between observers, for which they apply McNemar’s *χ*^2^ test (Kirkwood & Sterne, 2003). In other situations, concerned with quantification of effect intensities of potential harmful or protective expositions to the increasing, decreasing, or mere existence of diseases at the populational level, odds ratio estimations are traditionally applied (King, Harper, & Young, 2012).

In all these frequent situations, dichotomization is handy. Sometimes it is imposed by the study design when a researcher is compared to another one or when patients are classified into two levels (e.g., male or female). In other cases, some longed-evaluated cutoff is determined and applied to label two groups as healthy or diseased, exposed or non-exposed, showing a given effect or not. It is not a defect of scientific research, but the recommended most parsimonious approach to start an investigation with the simplest classification of events. Statistics has the role to refine researcher impressions, providing evidence for or against initial hypotheses. Therefore, it must be useful if statistical procedures are reliable in several situations .

Before one alleges that this study is approaching extreme, atypical, or unrealistic scenarios, let us consider that imbalanced tables are the main goal for researchers. Psychologists desire the highest agreement between assessors while clinicians expect the strongest association between intervention and patient outcome, thus resulting in 2x2 tables with data concentration along the main or off-diagonal. Likewise, epidemiologists pursue situations of high association between exposition and effect on populational diseases, usually low in prevalence, resulting in 2x2 tables with relatively empty cells corresponding to affected people.

Conversely, balanced tables improve little scientific knowledge for they would show the inability to obtain consistent measures from a given method, absence of treatment effect, or lack of relationship between exposition and effect. No one starts a scientific study unless the hypothesis is solid enough and the method is designed to control confusing variables. When, after all the best researcher efforts, the 2x2 table results are well balanced, statistical tests will show non-rejection of the null hypothesis and the study failed to confirm one’s expectation, remaining dubious conclusions either by lack of effect or sample insufficiency.

These few examples show that imbalanced tables are the rule, not the exception. Estimators for agreement or disagreement between observations or observers (generically named as ‘raters’ along this text) must be robust, therefore, to extreme tables in order to provide inferential statistics support to the researchers.

Agreement coefficients are fundamental statistics. Except for Francis Galton and Karl Pearson starting in the 1880s, who created the correlation coefficient (without the intention to apply it to assess agreement, although the computation of this work shows its equivalence) and the pioneering work of Yule, 1912 (who created *Q* and *Y* coefficients, explicitly intending to measure the association between nominal variables), there was great interest and creation of agreement coefficients between the 1940s and 1970s (Dice, 1945; Cramér, 1946; McNemar, 1947; Scott, 1955; Cohen, 1960; Holley & Guilford, 1964; Matthews, 1975; Hubert, 1977), including most of the coefficients in use nowadays. Renewed interest appears in this century, with the creation of new coefficients, searching for improvement and avoidance of known flaws of the older propositions (Gwet, 2008, 2010; Shankar & Bangdiwala, 2014), or attempts to improve traditional estimators (e.g., Lu, 2010, Lu, Wang, & Zhang, 2017).

The two most used coefficients explored here are the widespread and famous Cohen’s *κ*, which was the departure point of the present work, and McNemar’s *χ*^2^, praised in Epidemiology textbooks (Kirkwood & Sterne, 2003)(p. 218). Here we exhaustively analyzed all possible tables with total sizes from 1 to 68, totaling 1,028,789 different contingency tables to create a comprehensive map of several traditional association or agreement coefficients. By ‘exhaustive’ we mean the computation of all possible configurations of 2x2 tables, given the number of observations. The range matters to cover different qualitative scenarios (see “Methods, Table range”).

This study includes Cohen’s *κ*, Holley and Guilford’s *G*, Yule’s *Q*, Yules’s *Y*, Pearson’s *r*, McNemar’s *χ*^2^, Scott’s *π*, Krippendorff’s *α*, Dice’s *F*1, Shankar and Bangdiwala’s *B*, and Gwet’s *AC*1. We found that some estimators may compute improperly with imbalanced tables due to higher counts concentrated in one or more table cells, that the traditional Cohen’s *κ* and McNemar’s *χ*^2^ are problematic, and that *G* and *AC*1 are the best agreement coefficients.

## Methods

### Notation

To simplify mathematical notation along this text, a convention was adopted according to Table 1. *A* and *B* are representations of events (positive observer evaluation, existence of disease, exposition or effect, etc.), and $\bar {A}$ and $\bar {B}$ are their respective negations (negative observation, health individuals, absence of exposition or effect, etc.). In the main diagonal, *a* and *d* are counts or proportions of positive and negative agreements. In the off-diagonal, *b* and *c* are counting or proportions of disagreements. The total sample size along this text is *n* = *a* + *b* + *c* + *d*.

**Table 1:** Convention for 2x2 tables

	*B*	$\bar {B}$	
*A*	*a*	*b*	*a* + *b*
$\bar {A}$	*c*	*d*	*c* + *d*
	*a* + *c*	*b* + *d*	*a* + *b* + *c* + *d*

### Table range

All estimators selected below were exhaustively studied with tables from 1 to 68 observations, totaling 1,028,789 tables. Each of these tables is different, containing all possible configurations of *a*, *b*, *c*, *d* given the total number of observations, *n*. For instance, with *n* = 1 there are four possible tables, with *n* = 2 there are ten possible tables, and with 68 tables there are 57,155 possible tables.

This range is important because it covers small to large samples, comfortably surpassing some traditional cutoff sizes (e.g., *n* > 15 for exact Fisher test, *n* > 20 for Pearson’s *χ*^2^, *n* > 20 for Spearman’s *ρ*, *n* > 35 for McNemar’s *χ*^2^), beyond which asymptotic tests and the central limit theorem is valid (Siegel & Castellan, 1988). Simulations and essential estimators presented here were implemented and provided to allow replication of our findings in R, a free statistical language (R Core Team, 2022).

### Cohen’s kappa

This test was originally published by Cohen in (1960). The original publication proposed a statistical method to verify agreement, typically applied to comparison of observers or results of two measurement methods such as laboratory results (raters). Kappa (*κ*) statistics is given by

1 $$\kappa = \frac{p_{o} - p_{c}}{1 - p_{c}}$$

where *p*_*o*_ is the realized proportion of agreement and *p*_*c*_ is the expected proportions by chance (i.e., under assumption of null hypothesis) in which both raters agreed (i.e., the sum of proportions along the matrix main diagonal).

For 2x2 tables, in more concrete terms,

2 $$\begin{array}{@{}rcl@{}} && p_{o}={\frac{a+d}{n}}\\ &&p_{c}={\frac{(a+b)(a+c)+(c+d)(b+d)}{n^{2}}} \end{array}$$

Thus, Cohen’s *κ* can be computed, showing tension between the main (*ad*) and off (*bc*) diagonals, by

3 $$\kappa = {\frac{2(ad-bc)}{(a+c)(c+d)+(b+d)(a+b)}}$$

Historically, the first contingency table presented in the seminal paper of Cohen (1960), shows two clinical psychologists classifying individuals into schizophrenic, neurotic, or brain-damaged categories. This table is an example of a 3x3 table with proportions of agreement or disagreement between raters, while researchers more often pursue agreement in 2x2 tables. It follows a lengthy discussion of traditional measures of agreement such as Pearson’s *χ*^2^ and contingent coefficient. Curiously, for this first contingency table, Cohen only computed *χ*^2^ and reported it as significant, concluding by the existence of association but arguing that *χ*^2^ is not a defensible measurement of agreement. However, in 2x2 tables both association and agreement are coincident and provide equal *p* values, thus Pearson’s *χ*^2^ and Cohen’s *κ* are equivalent (Feingold, 1992), which may suggest that, at least in 2x2 tables, Cohen’s *κ* is a mere test of association. The computation of *κ* was not presented in Cohen’s paper for this first 3x3 table, thus we computed *κ* = − 0.092, concluding that this is an example of slight disagreement between the raters, an unfortunate initial example for who is presenting a new measure of agreement. It follows the introduction of the *κ* calculation, mentioning other similar statistics such as Scott’s *π*, which was published in 1955 (Scott, 1955). Then a second contingency 3x3 table is presented, bringing the same storyline of two psychologists classifying patients into three categories, for which the numbers were subtly amended, increasing two out of three values in the main diagonal and decreasing five out of six values in the other table cells to compute *χ*^2^ and *κ* in a scenario of rater’s agreement.

In addition, when the intensity of agreement is to be qualified, Cohen’s recommendation is to compute kappa statistics maximum value, *κ*_*M*_, permitted by the marginals to correct the agreement value of *κ* by *κ*/*κ*_*M*_, which was largely forgotten in the literature (Sim & Wright, 2005). The maximum *κ* is given by:

4 $$\kappa_{M} = \frac{p_{oM} - p_{c}}{1 - p_{c}}$$

where *p*_*oM*_ is the sum of minimal marginal values taken in pairs. For 2x2 tables it is

5 $$p_{oM} = min\left((a+c),(a+b) \right) + min\left((b+d),(c+d) \right)$$

Cohen did not propose correction by any lower bound when *κ* < 0 (i.e., rater disagreement), stating that it is more complicated and depends on the marginal values. For this lower limit of *κ*, we quote:

“The lower limit of K is more complicated since it depends on the marginal distributions. [...] Since *κ* is used as a measure of agreement, the complexities of its lower limit are of primarily academic interest. It is of importance that its upper limit is 1.00. If it is less than zero (i.e., the observed agreement is less than expected by chance), it is likely to be of no further practical interest.” (Cohen, 1960)

From this consideration, we have decided not to attempt any correction for negative values of *κ*. Along this text we refer to Cohen’s *κ* normalized, when *κ* > 0, as “Cohen’s *κ* corrected by *κ*_*M*_.” After this correction, the intensity of *κ* can be defined by Table 2, but there is an additional complication for that criteria varies according to different authors (Wongpakaran, Wongpakaran, Wedding, & Gwet, 2013).

**Table 2:** Criteria for qualitative categorization of *κ* according different authors – (modified from Wongpakaran et al., 2013)

*κ*	Landis and Koch	*κ*	Altman	*κ*	Fleiss
[-1.0,0.0)	Poor				
[0.0,0.2)	Slight	[-1.0,0.2)	Poor		
[0.2,0.4)	Fair	[0.2,0.4)	Fair	[-1.0,0.4)	Poor
[0.4,0.6)	Moderate	[0.4,0.6)	Moderate		
[0.6,0.8)	Substantial	[0.6,0.8)	Good	[0.4,0.75)	Intermediate to good
[0.8,1.0]	Almost perfect	[0.8,1.0]	Very good	[0.75,1.0]	Excellent

### Concurrent agreement measures

Many other alternative statistics have been proposed to compute agreement, although not always initially conceived for this purpose. Besides Cohen’s *κ*, here we selected Holley and Guilford’s *G*, Yule’s *Q*, Yules’s *Y*, Pearson’s *r*, McNemar’s *χ*^2^, Scott’s *π*, Dice’s *F*1, Shankar and Bangdiwala’s *B*, and Gwet’s *AC*1.

Some other estimators are redundant and were not analyzed for varied reasons:

- Janson and Vangelius’ *J*, Daniel–Kendall’s generalized correlation coefficient, Vegelius’ E-correlation, and Hubert’s *Γ* (see “Holley and Guilford’s *G*”);
- Goodman–Kruskal’s *γ*, odds ratio, and risk ratio (see “Yule’s Q”);
- Pearson’s *χ*^2^, Yule’s *ϕ*, Cramér’s *V*, Matthews’ correlation coefficient, and Pearson’s contingent coefficient (see “Cramér’s V” and “Pearson’s *r*”);
- Fleiss’ *κ* (see section “Scott’s *π*”).

All these alternatives are effect-size measures, therefore independent of sample size, *n*. A brief description of each test in the present context follows.

#### Holley and Guilford’s *G*

One of the simplest approaches to a 2x2 table was proposed by Holley & Guilford (1964), given by

6 $$G = \frac{(a+d)-(b+c)}{a+b+c+d}$$

A generalized agreement index is *J* (Janson & Vegelius, 1982) that can be applied to larger tables. However, in 2x2 tables it reduces to *J* = *G*^2^, thus *J* performance was excluded from the current analysis.

Other proposed coefficient, Hubert’s *Γ* (1977) is a special case of Daniel–Kendall’s generalized correlation coefficient and Vegelius’ E-correlation (Janson & Vegelius, 1982). For 2x2 tables it is computed by

7 $${{\varGamma}} = { 1 - 4 \frac{(a+d)(b+c)}{n^{2}}}$$

Although it looks like another coefficient, it is possible to show its equivalency to:

8 $${{\varGamma}} = {\left({\frac{ (a+d)-(b+c)}{a+b+c+d}} \right)^{2} } = G^{2}$$

Since it is redundant to both *J* and Holley and Guilford *G*, the analysis of the *Γ* coefficient is also not required.

According to Zhao, Liu, & Deng (2013), *G* index was independently rediscovered by other authors. In special, it was preceded by Bennett, Alpert, & Goldstein (1954), *S* which is computed by

9 $$S = \frac{k}{k-1} \left(P - \frac{1}{k} \right)$$

where *k* is the number of categories. Being *k* = 2 and ${P = \frac {a+d}{a+b+c+d}}$ in 2x2 tables, from Eq. 9 it is derived that:

10 $$S = \frac{(a+d)-(b+c)}{a+b+c+d}$$

Therefore, *S* is equal to *G*. However, here we kept the credit to Holley and Guilford’s *G* for historical reasons. The literature attributes this measure to the last authors. While Bennett et al. (1954) were chiefly focused on the question of consistency and presented it in the form of Eq. 9, Holley and Guilford extensively divulged and applied this index in terms of inter-rater reliability, being the first authors to formulate this coefficient for 2x2 tables in terms of *abcd* presented in Eq. 6.

In addition to that, Lienert (1972) proposed an inferential asymptotic statistical test for Holley & Guilford (1964) *G*, computing:

11 $$u = \frac{a+d-\frac{n}{2}}{\sqrt{\frac{n}{4}}}$$

For large samples (*n* > 30), the statistic (*a* + *d*) has distribution approximately normal with mean $\frac {n}{2}$ and variance $\frac {n}{4}$; consequently, *u* has standard *z* distribution, from which we can compute the correspondent two-sided *p* values to the statistical decision under the null hypothesis, *H*_0_ : *G* = 0. This inferential decision became necessary for the present study because this coefficient was elected as the benchmark for reasons exposed under the Section “Results”.

#### Yule’s Q

Goodman–Kruskal’s *γ* measures the association between ordinal variables. In the special case of 2x2 tables, Goodman–Kruskal’s *γ* corresponds to Yule's *Q* (1912), also known as Yule’s coefficient of association. It can be computed by

12 $$Q = \frac{ad-bc}{ad+bc}$$

Yule’s *Q* is also related to odds ratio, which was not conceived nor applied as an agreement measure (although it could be). The relationship is

13 $$OR = \frac{ad}{bc} = \frac{1+Q}{1-Q}$$

It is recommended to express *OR* as a logarithm. Therefore,

14 $$log(OR) = log(ad)-log(bc)$$

Again, it is possible to observe that *OR* and *Q* are statistics from the same tension-between-diagonals family.

Risk ratio (*RR*) also belongs to this family, being defined (assuming exposition in rows and outcome in columns of a 2x2 table) as the probability of outcome among exposed individuals relative to the probability of outcome among non-exposed individuals. Since *RR* describes only probability ratio of occurrence of outcomes, we propose to define it as a positive risk ratio, computed by

15 $$RR_{+} = \frac{\frac{a}{a+b}}{\frac{c}{c+d}}$$

To our knowledge, it is not usual in epidemiology the definition of a negative risk ratio (the ratio between the probabilities of absence of outcome among exposed individuals and absence of outcome among non-exposed individuals), which should be conceived as

16 $$RR_{-} = \frac{\frac{b}{a+b}}{\frac{d}{c+d}}$$

Consequently:

17 $$\begin{array}{@{}rcl@{}} OR = \frac{RR_{+}}{RR_{-}} = \frac{ad}{bc} \end{array}$$

Therefore, it is arguable that the traditional *RR*_+_ is a somewhat incomplete measure of agreement, for it does not explore all the information of a 2x2 table when compared to *OR*.

Both *RR* and *OR* are transformations of *Q*, inheriting their characteristics. For that reason, only *Q* is analyzed in this work.

#### Yules’s *Y*

The coefficient of colligation, *Y*, was also developed by Yule (1912). It is computed by

18 $$Y = \frac{\sqrt{ad}-\sqrt{bc}}{\sqrt{ad}+\sqrt{bc}}$$

which is a variant of Yule’s *Q*. Here, each term can be interpreted as a geometric mean.

#### Cramér’s V

The traditional Pearson’s chi-squared (*χ*^2^) test can be computed by

19 $$X^{2}= \frac{(ad-bc)^{2}(a+b+c+d)}{(a+b)(c+d)(a+c)(b+d)}$$

This formulation is interesting to reveal *χ*^2^ statistics containing tension between diagonals, *ad* − *bc*.

For the special case of 2x2 tables, absolute value of *κ* and *χ*^2^ are associated (Feingold, 1992). However, it is observed that *χ*^2^ statistics is not an effect size measurement because it depends on sample sizes thus, in its pure form, *χ*^2^ does not belong to the agreement-family of coefficients. In order to remove sample size dependence and turn *χ*^2^ statistics into an effect size measurement, it should be divided by *n* = *a* + *b* + *c* + *d*. The squared root of this transformation is Cramér’s *V* (1946), computed by

20 $$V = \sqrt{\frac{X^{2}}{n}} = \frac{|ad-bc|}{\sqrt{(a+b)(c+d)(a+c)(b+d)}}$$

Cramér’s *V* can also be regarded as the absolute value of an implicit Pearson’s correlation between nominal variables or, in other words, an effect size measure ranging from 0 to 1. Cramer’s *V*, in other words, is the tension between diagonals, *ad* − *bc* (which is the 2x2 matrix determinant) normalized by the product of marginals (*a* + *b*) (*c* + *d*) (*a* + *c*) (*b* + *d*).

#### Pearson’s *r*

There are many relations and mathematical identities among coefficients that converge to Pearson’s correlation coefficient, *r*.

Matthews’ correlation coefficient is a measure of association between dichotomous variables (Matthews, 1975) also based on *χ*^2^ statistics. Since it is defined from the assessment of true and false positives and negatives, it is regarded as a measure of agreement between measurement methods. It happens that Matthews’ correlation coefficient is identical to Pearson’s *ϕ* coefficient and Yule’s *ϕ* coefficient (Cohen, 1968), computed by

21 $$\phi = \frac{ad-bc}{\sqrt{(a+b)(a+c)(b+d)(c+d)}}$$

Another famous estimator is the Pearson’s contingent coefficient, usually defined from chi-squared statistics and also expressed as function of *ϕ* by

22 $$PCC = \sqrt{\frac{X^{2}}{X^{2}+n}} = \sqrt{\frac{\phi^{2}}{\phi^{2}+1}}$$

It was not included in this analysis for two reasons: it is not taken as an agreement coefficient and its value ranges from zero to $\sqrt {\frac {1}{2}}\approx 0.707$, which makes this coefficient not promising to the current context.

Other two correlation coefficients, Spearman’s *ρ* and Kendall’s *τ*, also provide the same values of Pearson’s *r* for 2x2 tables. In the notation adopted here:

23 $$r = \rho = \tau = \frac{ad-bc}{\sqrt{(a+b)(a+c)(b+d)(c+d)}}$$

From Eq. 23 other coincidences are observed:

- Equation 21 shows that *ϕ* = *r* for 2x2 tables, thus Matthews’ correlation coefficient and Pearson’s contingent coefficient also share *r* properties.
- Equation 20 shows that Cramér’s *V* merely is the absolute value of Pearson’s *ϕ* coefficient (Matthews, 1975), which is, in turn, equal to *r*.

Consequently, for the current work, only Pearson’s *r* is computed as representative of all these other estimators.

#### McNemar’s chi-squared

This test was created by McNemar (1947), and became known in the literature as McNemar’s *χ*^2^. It is applicable to 2x2 tables by

24 $$X^{2}_{MN}={\frac{(b-c)^{2}}{b+c}}$$

to assess marginal homogeneity. A typical example is verification of change before and after an intervention, such as the disappearance of disease under treatment in a given number of subjects.

The inferential test is based on the probability ratio and confidence interval given by $\frac {b}{c}$; the null hypothesis is rejected when the unitary value is not included in its confidence interval 95%. In other words, McNemar’s *χ*^2^ resembles an odds ratio but it is incomplete for not including the main diagonal.

This traditional McNemar’s *χ*^2^ cannot be directly confronted with other estimators because it is not restricted to the interval [-1,1]. It can be normalized to show values between 0 and 1 as an effect-size measurement if divided by |*b* − *c*|, resulting in

25 $$MN={\frac{\frac{(b-c)^{2}}{b+c}}{|b-c|}}={\frac{|b-c|}{b+c}}$$

It is noteworthy to say that both McNemar’s *χ*^2^ and its normalized correspondent *MN* are even more partial than *RR*, for they use only the information of the off-diagonal. Problems caused by such weakness are explored below.

Two recent reviews aimed to include information from the main diagonal to improve the traditional McNemar’s *χ*^2^ (Lu, 2010; Lu et al., 2017). The first review of McNemar’s *χ*^2^ (Lu, 2010) computes:

26 $$X^{2}_{MN (2010)}={\frac{(b-c)^{2}}{1 + \frac{a+d}{a+b+c+d}}}$$

To show the obtained improvement, this author applied Pearson’s *χ*^2^ statistics to compare five exemplary and exact binomial test by fixing the *b* and *c* and varying *a* + *d*.

The second review (Lu et al., 2017) aimed a further improvement in order to differentiate situation in which *a* and *d* dominates the main diagonal, by proposing:

27 $$X^{2}_{MN (2017)}={\frac{(a+b+c+d)(b-c)^{2}}{(2a+b+c)(2d+b+c)}}$$

Following the same strategy, these authors compared Pearson’s *χ*^2^ statistics of several tables with fixed two values of *b* and *c* and varied values of *a* and *d* trying to show the improvement of this new equation.

Since that, to our knowledge, there is no implementation of these methods in R packages with inferential statistics, we may apply bootstrapping (Efron, 2007) to reject the null hypothesis when *X*^2^ = 0 does not belong to the 95% prediction HDI interval (see “Analysis methods” for details).

#### Scott’s *π*

This statistics is similar to Cohen’s *κ* to measure inter-rater reliability for nominal variables (Scott, 1955). It applies the same equation of *κ* but it changes the estimation of *p*_*c*_ using squared joint proportions, computed as

28 $$\pi = { \frac{p_{o} - p_{c}}{1 - p_{c}} }$$

where

29 $$p_{c} = \left({\frac{a+c+a+b}{2 n}} \right)^{2} + \left({\frac{c+d+b+d}{2 n}} \right)^{2}$$

thus

30 $$\pi = \frac{{ad-({\frac{b+c}{2}}} )^{2}}{({a+ {\frac{b+c}{2}}})({d+ \frac{b+c}{2}})}$$

Besides the tension between the diagonals shown on the numerator of this expression (*ad* vs. *b* + *c*), Scott’s *π* also coincides with Fleiss’ *κ* in the special case of 2x2 tables, thus our analysis is restricted to Scott’s *π*.

There is also Krippendorff’s *α* (1970). It converges, at least asymptotically, to Scott’s *π* in 2x2 tables with nominal categories and two raters (Wikipedia, 2021). The structure of several other coefficients may be unified in a single structure comparing the observed and expected proportions or probabilities (Feng, 2015). This generalized structure, from which Krippendorff’s *α* is part of the family (Gwet, 2011; Krippendorff, 2011), may become equivalent to different coefficients of the so-called *S* family (including Bennett’s *S*, Holley and Guilford’s *G*, Cohen’s *κ*, Scott’s *π*, and Gwet’s *AC*1), depending on what assumptions are adopted for the chance agreement (Feng, 2015; Krippendorff, 2016; Feng & Zhao, 2016). This coefficient deals with any number of categories or raters (Krippendorff, 2011) and is provided by a combinatory computational procedure (Gwet, 2011; Krippendorff, 2011). Although it was not possible, so far, to deduce a feasible general *abcd*-formula, it was tested with the implementation by Hughes (2021) to verify if its pattern is close to Scott’s *π*, as predicted by theory, for 2x2 tables with nominal categories (see Supplemental Material, “Implementation of Krippendorff’s *α*” for details).

#### Dice’s *F*1

Also known as F-score or F-measure, it was first developed by Dice (1945) as a measure of test accuracy, therefore it can be assumed as an agreement statistics in the same sense as the Matthews’ correlation coefficient described above.

It is computed by

31 $$F1 = { \frac{a}{a+{\frac{b+c}{2} }} } = \frac{2a}{2a+b+c}$$

*F*1 has been suggested to be a suitable agreement measure to replace Cohen’s *κ* in medical situations (Hripcsak & Rothschild, 2005), thus its analysis was included here.

There are fundamental differences between *F*1 and all other agreement statistics. It does not belong to the tension-between-diagonals family, for it computes only the proportion between positive agreement (*a*) and the upper-left triangle of a 2x2 table.

In addition, *F*1 is difficult to compare a priori with other measurements because it ranges from *F*1 = 0 if *a* = 0 (disagreement) to *F*1 = 1 if *b* = 0 and *c* = 0 (agreement) in both cases neglecting agreements in negative counts (*d*). Being the range of *F*1 shorter than that of other measurements, neutral situations (i.e., when there is no agreement nor disagreement) should find *F*1 ≈ 0.5, while the concurrent measurements presented here should provide zero. In order to make its range more comparable, we propose to rescale to the interval [− 1,1] given by

32 $$F1_{adj} = { 2F1 - 1} = \frac{2a-(b+c)}{2a+(b+c)}$$

which adjusts *F*1 range to span from -1 to 1 without changing its original pattern. Interestingly, this rescaling created a partial tension between the positive agreement and the off-diagonal that was not present in *F*1 original presentation.

#### Shankar and Bangdiwala’s *B*

This coefficient has proposed this statistics to access 2x2 tables, reporting its good performance (Shankar & Bangdiwala, 2014). This statistics is computed by

33 $$B = \frac{a^{2}+d^{2}}{(a+c)(a+b)+(b+d)(c+d)}$$

Similar to Dice’s *F*1, this estimator ranges from 0 to 1, being 0 correspondent to disagreement, 0.5 to neutrality, and 1 to agreement. Therefore, we also propose to explore its adjustment by scaling to the range [− 1,1] with:

34 $$B_{adj} = 2B-1 = {\frac{a^{2}+d^{2}- \left(2bc+(a+d)(b+c) \right)}{a^{2}+d^{2}+ \left(2bc+(a+d)(b+c) \right) }}$$

#### Gwet’s *AC*1

This first-order agreement coefficient (*AC*1) was developed by Gwet (2008) as an attempt to correct Cohen’s *κ* distortions when there is high or low agreement. *AC*1 seems to have better performance than Cohen’s *κ* assessing inter-rater reliability analysis of personality disorders (Wongpakaran et al., 2013; Xie, Gadepalli, & Cheetham, 2017) and has been applied in information retrieval (Manning, Raghavan, & Schutze, 2008).

It is computed by

35 $$AC1 = { \frac{a^{2}+d^{2}- {\frac{(b+c)^{2}}{2}} }{{ a^{2} + d^{2} + {\frac{(b+c)^{2}}{2}}} + (a+d)(b+c)} }$$

*AC*1 somewhat reflects the tension between diagonals, since there is added values for *a* and *d* and subtracted values of *b* and *c* in the numerator.

### Association and agreement

Part of the problem is to define what is really being measured by any of these coefficients. Lienert (1972) argued that association tests are not of the same nature as tests for agreement. Association tests the null hypothesis

36 $$H_{0,~ind}:(\alpha \cdot \delta) - (\beta \cdot \gamma) = 0$$

while agreement assesses

37 $$H_{0,~agr}:(\alpha+\delta)-(\beta+\gamma) = 0$$

where *α*, *β*, *γ*, and *δ* are the populational proportions respectively estimated by *a*, *b*, *c*, and *d* (see notation on Table 1). Association tests (from which Cohen’s *κ*, Pearson’s *ϕ* and other Pearson’s *χ*^2^-based statistics are representatives), and agreement tests (from which *G* is a representative) are, therefore, sensitive to different types of association (Shreiner, 1980).

In the same line of reasoning, Pearson’s *χ*^2^ and contingent coefficient, as well McNemar test (McNemar, 1947), were previously criticized by Cohen (1968) who stated that association does not imply necessarily in agreement for any table size. This aspect will be further discussed below. The question is to know how these coefficients are related, when they measure association or agreement, and when their measures are coincident or discrepant.

Holley and Guilford (1964) showed that *G* is equal to Pearson’s *ϕ* coefficient only when the marginal values, i.e., $\frac {a+b}{n} = \frac {a+c}{n} = \frac {b+d}{n} = \frac {c+d}{n} = 0.5$ where *n* = *a* + *b* + *c* + *d*, a condition in which *G* = *ϕ* = 0 but also *κ* = 0. It is to say that *κ* and *ϕ* are, otherwise, different entities of *G*, with potential different performances to detect association or agreement in 2x2 contingency tables: *ϕ* and *G* are related (Holley & Guilford, 1964) by

38 $$\phi = \frac{ \frac{G}{4} - \left(\frac{a+b}{n} - \frac{1}{2} \right) \left(\frac{a+c}{n} - \frac{1}{2} \right) }{ \sqrt{\frac{a+b}{n} ~ \frac{a+c}{n} ~ \frac{b+d}{n} ~ \frac{c+d}{n}} }$$

and *κ* is related to *G* (Green, 1981) by

39 $$\kappa = {\frac{ \frac{G+1}{2} - p_{c} }{1-p_{c}}}$$

The parcel *p*_*c*_, included in the computation of *κ*, is known in the literature as the ‘chance correction factor’. Since *κ* includes more parcels, one would expect that the performance of *κ* should exceed that of *G*. However, Green (1981) stated that “Because the standard equation for kappa clearly includes a chance correction factor, many authors [...] have suggested its usage. Unfortunately, chance has not been explicitly defined.” It was also shown that, under skewed marginals, *κ* and *ϕ* underestimate agreement, while *G* is a stable estimator (Shreiner, 1980).

### Analysis methods

In order to assess the performance of different agreement coefficients, first we developed the comparative analysis of all these statistics by challenging them with fabricated 2x2 tables, considering scenarios with more balanced tables, and then with more extreme tables containing 0 or 1 in some cells. These analyses confront intuition of agreement and the measures among all estimates (see “Results, Challenge tables”).

A second step is the analysis of all possible tables with *n* = 64 (see section “Inferential statistics: tables with *n* = 64” for details), verifying the coincidence and stability of statistical decisions taken from the estimators. For that, we developed a R function, agr2x2_gentablen(n), which can create all combinations of 2x2 tables with *n* observations. This function and examples of its use are available in the Supplemental Material (see “Creating all 2x2 tables with size *n*”). To compare the performance of estimators, not only the estimate of the agreement coefficients but also the decision by inferential statistics is required. Thus, inferential statistics for all proposed estimators applying R functions from selected packages were computed when available. When there was no function available, the confidence interval was computed by bootstrapping (this procedure is described for the simple agreement coefficient, *SAC*, in the Supplemental Material, see “Implementation of Holley and Guilford’s *G*” using the results of Bell and Kato-Katz examination, Hoff et al. (1982)). Exhaustive testing showed that Holley and Guilford’s *G*, among all the studied estimators, minimized the discrepancy of inferential decisions from the others, and was selected as benchmark (see “Figures and performance checking” for details).

Finally, in “Comprehensive maps: all tables with 1 ≤ *n* ≤ 68” we exhaustively mapped all estimates from all possible tables created in this range of sizes to show the entire region that each concurrent estimator covers. Tables with size ranging from 1 to 68 were generated (see Supplemental Material “Creating all 2x2 tables with size *n*”), which resulted in a little more than 1 million (exactly 1,028,789) different 2x2 tables covering all possible configurations of ‘*abcd*’. A global measurement of estimator qualities was computed by the Pearson’s and Spearman’s correlations between Holley and Guilford’s *G* and other estimators across all 1,028,789 tables. Pearson’s correlation (not to be confounded with Pearson’s *r* application to the agreement coefficient under investigation here) assesses linear trend, while Spearman’s assesses the monotonic trend of each pair of estimators. These correlations were separately estimated for each *n* (from 1 to 68) and, then, lower (HDI LB) and upper (HDI UB) bounds of the 95% highest density interval (HDI95%) were obtained (see Supplemental Material, “Computation of Table 6”).HDI 95% is the smaller prediction interval that contains 95% of the probability density function (Hyndman, 1996).

All R source codes and data presented in this work, and some others not shown in the main text are hosted by Harvard Dataverse 10.7910/DVN/HMYTCK

## Results

### Challenge tables

A set of 17 tables was chosen to represent several scenarios: eight of agreement, two of neutrality and seven of disagreement in several 2x2 configurations (Tables 3 and 4).

In Table 3, regular scenarios were tested with three levels of agreement, three levels of disagreement, two neutral scenarios, and a parallel scenario were *b* = 2*a* and *d* = 2*c*. The intention here is to confront one’s intuition with values provided by all estimators assessed in the current work.

**Table 3:** Test performance of estimators with varied scenarios offering 2x2 regular tables; texts in bold indicate discrepant estimates, *f* / *t* is counting of discrepancies (failures) over the scenarios (trials)

		agr.high	agr.high	agr.low	dis.high	dis.high	dis.low	neutral	neutral	dis. a/c=b/d
	*f* / *t*	$$\left [ \begin {array}{cc} 90 & 10 \\ 10 & 90 \end {array}\right ]$$	$$\left [ \begin {array}{cc} 90 & 11 \\ 9 & 90 \end {array}\right ]$$	$$\left [ \begin {array}{cc} 60 & 41 \\ 39 & 60 \end {array}\right ]$$	$$\left [ \begin {array}{cc} 10 & 90 \\ 90 & 10 \end {array}\right ]$$	$$\left [ \begin {array}{cc} 10 & 91 \\ 89 & 10 \end {array}\right ]$$	$$\left [ \begin {array}{cc} 41 & 60 \\ 60 & 39 \end {array}\right ]$$	$$\left [ \begin {array}{cc} 50 & 50 \\ 50 & 50 \end {array}\right ]$$	$$\left [ \begin {array}{cc} 75 & 25 \\ 75 & 25 \end {array}\right ]$$	$$\left [ \begin {array}{cc} 44 & 88 \\ 22 & 44 \end {array}\right ]$$
Holley and Guilford’s *G*	0/8	0.80000	0.80000	0.20000	–0.80000	–0.80000	–0.20000	0.00000	0.00000	–0.11111
Gwet’s *AC*1	1/9	0.80000	0.80000	0.20000	–0.80000	–0.80000	–0.19988	0.00000	**0.05882**	–0.11111
Scott’s *π*	1/9	0.80000	0.80000	0.20000	–0.80000	–0.80000	–0.20012	0.00000	**–0.06667**	–0.11111
Krippendorff’s *α*	1/9	0.80050	0.80050	0.20200	–0.79550	–0.79550	–0.19712	0.00250	**–0.06400**	–0.10831
Cohen’s *κ*	1/9	0.80000	0.80002	0.20008	–0.80000	–0.79982	–0.20012	0.00000	0.00000	**0.00000**
Cohen’s *κ* corrected by *κ*_*M*_	4/9	**1.00000**	**0.98000**	**0.98000**	–0.80000	–0.79982	–0.20012	0.00000	0.00000	**0.00000**
Pearson’s *r*	1/9	0.80000	0.80018	0.20012	–0.80000	–0.79998	–0.20012	0.00000	0.00000	**0.00000**
Yule’s *Q*	7/9	**0.97561**	**0.97585**	**0.38488**	**–0.97561**	**–0.97561**	**–0.38488**	0.00000	0.00000	**0.00000**
Yule’s *Y*	1/9	0.80000	0.80090	0.20015	–0.80000	–0.79999	–0.20015	0.00000	0.00000	**0.00000**
Shankar and Bangdiwala’s *B*,		0.81000	0.81008	0.36004	0.01000	0.01000	0.16008	0.25000	0.31250	0.22222
verified by rescaled *B*	9/9	**0.62000**	**0.62016**	**–0.27993**	**–0.98000**	**–0.98000**	**–0.67983**	**–0.50000**	**–0.37500**	**–0.55556**
Dice’s *F*1,		0.90000	0.90000	0.60000	0.10000	0.10000	0.40594	0.50000	0.60000	0.44444
verified by rescaled *F*1	1/9	0.80000	0.80000	0.20000	–0.80000	–0.80000	–0.18812	0.00000	**0.20000**	–0.11111
Normalized McNemar’s *χ*^2^	8/9	**0.00000**	**0.10000**	**0.02500**	**0.00000**	**0.01111**	**0.00000**	0.00000	**0.50000**	**0.60000**

It may be interesting to observe Table 3 by columns:

- This table indicates test names and counts of the number of problems to perform with the selected nine scenarios.
- Many tests provide a reasonable value for scenarios of high agreement, except Cohen’s *κ* corrected by *κ*_*M*_ (the maximum kappa, as recommended by the original author) and Yule’s *Q* (exaggerated values), Shankar and Bangdiwala’s *B* (underestimated when adjust is applied), and normalized McNemar’s *χ*^2^ (estimated as zero when *b* = *c* or underestimated when *b* and *c* are too close).
- The low agreement scenario shows what normalized McNemar’s *χ*^2^ underestimated while corrected Cohen’s *κ* and Yule’s *Q* overestimated values in comparison with other tests. Adjusted Shankar and Bangdiwala’s *B* mistakenly deemed this table as disagreement.
- The next three scenarios provide disagreement scenarios that are the reverse of the previous three. Yule’s *Q* reveals the same exaggeration for high disagreement (Cohen’s *κ* has no proposed correction for negative values). Normalized McNemar’s *χ*^2^ and Shankar and Bangdiwala’s *B* provide only positive values. If normalized McNemar’s *χ*^2^ is taken by its absolute number, disagreement was underestimated. Rescaled Shankar and Bangdiwala’s *B* shows that disagreement was overestimated.
- The first neutral scenario has all values equal. Raw Dice’s *F*1 is equal to 0.5 (as expected), providing zero when rescaled. Shankar and Bangdiwala’s *B*, however, could not deal with this completely neutral scenario, showing disagreement when rescaled.
- The next neutral scenario caused major problems for normalized McNemar’s *χ*^2^, Shankar and Bangdiwala’s *B* and Dice’s *F*1, while Scott’s *π* and Gwet’s *AC*1 slightly deviated from zero.
- The last scenario is a special situation of low disagreement that caused problems to many estimators. The matrix determinant of a parallel 2x2 table is null and all estimators belonging to the tension-between-diagonal family become, therefore, null. The slight disagreement was equally captured by *G*, Gwet’s *AC*1, Scott’s *π* and adjusted *F*1. Adjusted Shankar and Bangdiwala’s *B* and normalized McNemar’s *χ*^2^ overestimated the disagreement.

Table 4 shows a second set of unbalanced tables, with the presence of values 0 or 1 in some table cells to provide more extreme conditions, including scenarios of agreement or disagreement:

- Many estimators are problematic. Cohen’s *κ*, *κ* adjusted by maximum *κ*, Pearson’s *r*, Yule’s *Q* and *Y* present problems when there are zeros in some cells, providing null or non-computable estimates. *Q* and *Y* easily approached 1 or -1 even when the agreement or disagreement is not perfect. Normalized McNemar’s *χ*^2^ uses information only from the off-diagonal and it is disturbed when information is concentrated in the main diagonal. Despite its adjustment, Shankar and Bangdiwala’s *B* failed in some scenarios of disagreement.
- The second major cause of problems is due to 0 in the main diagonal (last four columns), leading to underestimation of agreement by Pearson’s *r*, Cohen’s *κ* and Scott’s *π*, and underestimation of disagreement by Pearson’s *r* and Cohen’s *κ*.
- When 0 appears in both diagonals (last two columns), many estimators are not computable while others produce underestimated values. Scott’s *π* underestimates agreement. Normalized McNemar’s *χ*^2^ overestimated agreement and disagreement. Holley and Guilford’s *G*, Gwet’s *AC*1, Dice’s *F*1, and Shankar and Bangdiwala’s *B* were able to generate adequate values in both scenarios.
- When there are no zeros (first three scenarios), still Yules’*Q* and *Y* may overestimate agreement of disagreement. Adjusted Shankar and Bangdiwala’s *B* underestimated agreement and overestimated agreement in the first two contingency tables. Normalized McNemar’s *χ*^2^ could not detect disagreement and agreement in second and third scenarios.

**Table 4:** Test performance of estimators with 2x2 challenge with extreme tables; texts in bold indicate discrepant estimates, *f* / *t* is counting of discrepancies (failures) over the scenarios (trials)

		agr. c = 1	dis. d = 1	agr. b = 1, c = 1	agr. b = 0, c = 1	agr. d = 0	dis. d = 0	agr. c = 0, d = 0	dis. c = 0, d = 0
	*f* / *t*	$$\left [ \begin {array}{cc} 94 & 11 \\ 1 & 94 \end {array}\right ]$$	$$\left [ \begin {array}{cc} 11 & 94 \\ 94 & 1 \end {array}\right ]$$	$$\left [ \begin {array}{cc} 99 & 1 \\ 1 & 99 \end {array}\right ]$$	$$\left [ \begin {array}{cc} 100 & 0 \\ 1 & 99 \end {array}\right ]$$	$$\left [ \begin {array}{cc} 180 & 10 \\ 10 & 0 \end {array}\right ]$$	$$\left [ \begin {array}{cc} 10 & 180 \\ 10 & 0 \end {array}\right ]$$	$$\left [ \begin {array}{cc} 190 & 10 \\ 0 & 0 \end {array}\right ]$$	$$\left [ \begin {array}{cc} 10 & 190 \\ 0 & 0 \end {array}\right ]$$
Holley and Guilford’s *G*	0/9	0.88000	–0.88000	0.98000	0.99000	0.80000	–0.90000	0.90000	–0.90000
Gwet’s *AC*1	0/8	0.88000	–0.87531	0.98000	0.99000	0.88950	–0.89526	0.94744	–0.89526
Scott’s *π*	2/8	0.88000	–0.88471	0.98000	0.99000	**–0.05263**	–0.90476	**–0.02564**	–0.90476
Krippendorff’s *α*	2/8	0.88030	–0.88000	0.98005	0.99002	**–0.05000**	–0.90000	**–0.02308**	–0.90000
Cohen’s *κ*	4/8	0.88030	–0.88471	0.98000	0.99000	**–0.05263**	**–0.10465**	**0.00000**	**0.00000**
Cohen’s *κ* corrected by *κ*_*M*_	4/8	0.90025	–0.88471	1.00000	0.99000	**–0.05263**	**–0.10465**	**0.00000**	**0.00000**
Pearson’s *r*	4/8	0.88471	–0.88471	0.98000	0.99005	**–0.05263**	**–0.68825**	**div/0**	**div/0**
Yule’s *Q*	6/8	**0.99751**	**–0.99751**	0.99980	1.00000	**–1.00000**	**–1.00000**	**div/0**	**div/0**
Yule’s *Y*	6/8	**0.93184**	**–0.93184**	0.98000	1.00000	**–1.00000**	**–1.00000**	**div/0**	**div/0**
Shankar and Bangdiwala’s *B*,		0.88581	0.00608	0.98010	0.99005	0.89503	0.01786	0.95000	0.05000
verified by rescaled *B*	3/8	**0.77163**	**–0.98783**	0.96020	0.98010	0.79006	**–0.96429**	0.90000	–0.90000
Dice’s *F*1,		0.94000	0.10476	0.99000	0.99502	0.94737	0.09524	0.97436	0.09524
verified by rescaled *F*1	0/8	0.88000	–0.79048	0.98000	0.99005	0.89474	–0.80952	0.94872	–0.80952
Normalized McNemar’s *χ*^2^	5/8	0.83333	**0.00000**	**0.00000**	1.00000	**0.00000**	0.89474	**1.00000**	**1.00000**

Based on this preliminary analysis, the famous Cohen’s *κ* failed in most extreme situations. Other indices that showed over and underestimation were unable to cope with disagreement or failed to generate a coherent value. The best estimators seem to be Holley and Guilford’s *G* and Gwet’s *AC*1. Scott’s *π* and Dice’s *F*1 are also competitive (since the rescaling of *F*1 makes possible the comparison with other coefficients).

### Inferential statistics: tables with *n* = 64

**Fig. 1:**
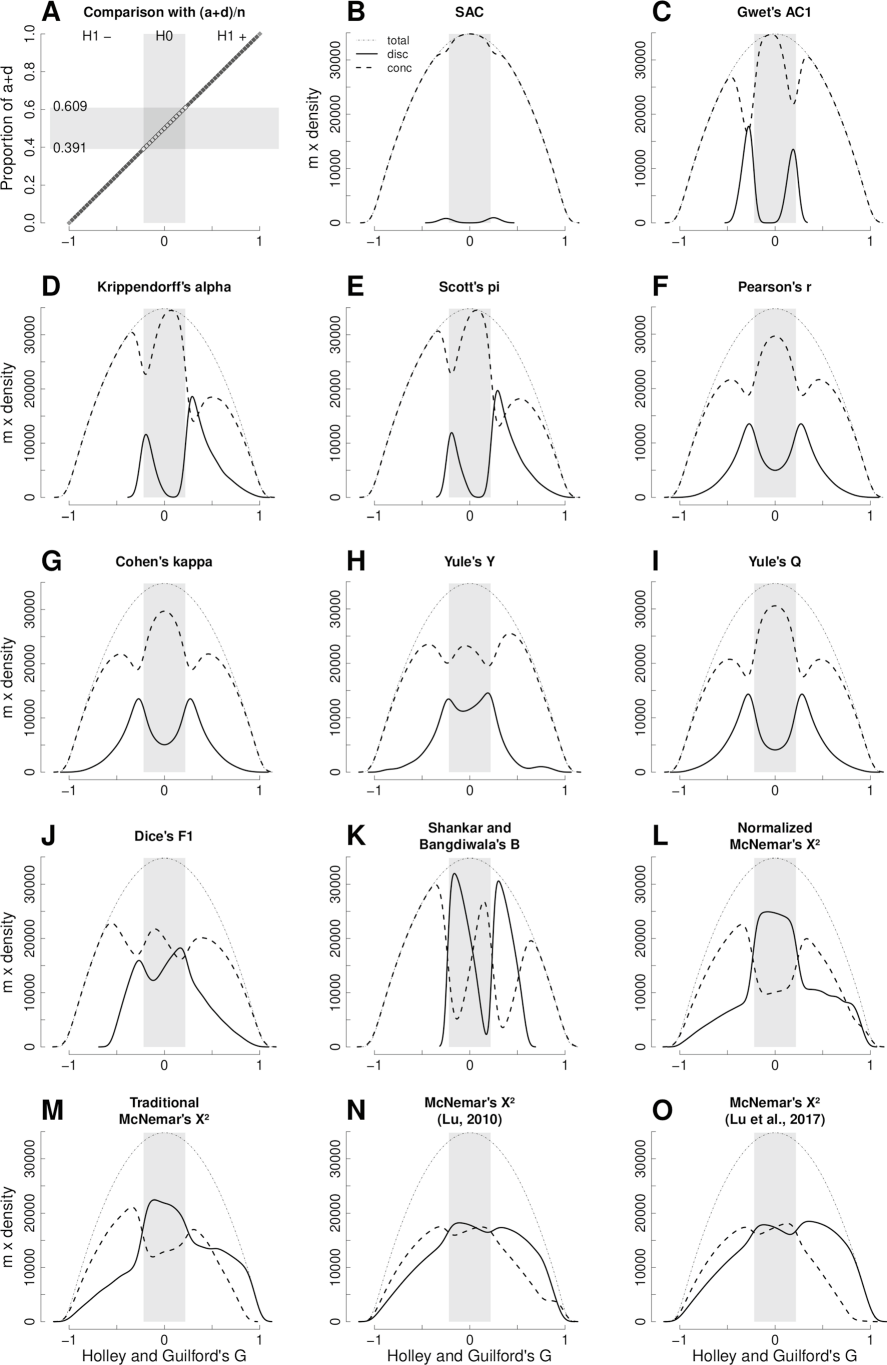
(**A**) Relative performance of proposed estimators concerning inferential statistics of Holley and Guilford’s coefficient (*G*). *G* is a linear transformation of (*a* + *d*)/*n*. The *gray area* corresponds to *p* ≥ 0.05. (**B** to **O**) probability distribution function of *G* multiplied by *m* tables: *lines* are the distribution of *G* from tables in which there is discrepancy (*solid lines*) or concordance (*dashed lines*) between the inferential decision of *G* (benchmark) and each other estimators. Mistakes are discrepancy of inferential decision and fails are impossibility of estimator computation (e.g., division by zero) provided as the proportion in relation to all 47,905 possible tables with n = 64

Figure 1 shows the computation of all possible 2x2 tables with 64 observations in function of Holley and Guilford’s *G*, which was selected as the benchmark (see “Analysis methods” in the Methods section and “Computation of Fig. 1 and Table 5” in the Supplemental Material for details). Both, the point-estimate and inferential decision of agreement or disagreement are compared, thus creating two probability density functions multiplied by the number of tables, *m* (i.e., the area under each curve is proportional to the number of involved tables). In comparison with other estimators, the solid lines show the distributions of *G* point-estimates of *G* computed from tables from which there is discrepancy in the rejection of the null hypothesis of absence of agreement; dashed lines are the same, in tables with concordance in this decision. Therefore, the greater the area under the solid line, the more the number of discrepancies between *G* and the agreement coefficient under assessment, which is counted as the percentage of total discrepancies and in three situations (Table 5): disagreement (*H*_1_ −), neutrality (*H*_0_), and agreement (*H*_1_ +).

Figure 1A differs from the other subfigures to show that Holley and Guilford’s *G* is perfectly correlated with the proportion $\frac {a+d}{n}$, thus representing the bisector of reference, which is another evidence that *G* can be a good choice for the benchmark. The interval proportion ${0.391 \le \frac {a+d}{n} \le 0.609}$ corresponds to the non-rejection of the null hypothesis, *H*_0_ : *G* = 0, interpreted here as populational neutrality (neither disagreement nor agreement, randomness). The other two regions denoted as H1- and H1+, correspond to the rejection of the null hypothesis, respectively meaning disagreement or agreement between raters. This corresponding interval of *G* appears in the subsequent panels (Fig. 1B to L). The total distribution of *G* has an ogival, symmetrical shape represented by a fine dotted line, which corresponds to the total of 47,905 possible tables with *n* = 64.

The small discrepancy on inferential statistics decision between *SAC* and *G* (Fig. 1B) is caused by differences between the bootstrapping and asymptotic statistical test (see Supplemental Material, “Implementation of Holley and Guilford’s *G*”); for this reason, a small number of discrepancies are located in the transition from H0 to H1 areas. Besides the number of total discrepancies (solid lines in Fig. 1), its location is also important:

- Gwet’s *AC*1 has no mistakes in H1+; mistakes in H1- are close to the transition to H0 (Fig. 1C).
- Krippendorff’s *α* and Scott’s *π* show similar patterns.
- Krippendorff’s *α* has a small number of discrepancies and Scott’s *π* has no discrepancies in H1- (Fig. 1D and E) - see also Table 5.
- Pearson’s *r* (Fig. 1F), Cohen’s *κ* (Fig. 1G), and Yule’s *Q* (Fig. 1I) are similar; the first two presented similar percentages of mistakes in all three areas, while the latter had fewer mistakes when rejecting the null hypothesis.
- Yule’s *Y* (Fig. 1H) was slightly better than Yule’s *Q* in the total number of discrepancies, but has more mistaken decisions around the null hypothesis.
- Dice’s *F*1 (Fig. 1J) presented a total number of discrepancies greater than Yule’s *Q* and asymmetry for the corresponding *G* distribution (regarding the inferential decision, Dice’s *F*1 and Dice’s *F*1 rescaled to the interval [-1,1] are identical, respectively checking if 0.5 and 0.0 are inside the estimated HDI95%).
- Similarly, original or rescaled Shankar and Bangdiwala’s *B* (Fig. 1K) are qualitatively similar to Scott’s *π* with quantitative worsened performance.
- Normalized McNemar’s *χ*^2^ (Fig. 1L) produced a flawed approach to the inferential statistics.
- Traditional McNemar’s *χ*^2^ (Fig. 1M) and the two recently revised (Lu, 2010; Lu et al., 2017) versions (Fig. 1N and O) do not generate values in the interval [-1,1] but, regarding inferential decisions are still comparable with *G*, showing increasing number of total discrepancies.

Table 5 shows counts of involved tables, errors and discrepancies from Fig. 1 generated with all 47,905 possible configurations of 2x2 tables with *n* = 64. Depending on table configuration, sometimes the estimator cannot be computed (failed estimator, e.g., by division by zero) or it is computed but the corresponding *p* value is not returned by R function (no *p* value). Otherwise, there is an inferential decision but discrepancies with the benchmark (Holley and Guilford’s *G*) may occur on the null hypothesis (when *G* does not reject and the other estimator rejects the null hypothesis) or on the rejection of the null hypothesis (*H*_1_ −, when *G* assumes disagreement but the other estimator does not reject the null hypothesis; *H*_1_ +, when *G* assumes agreement but the other estimator does not reject the null hypothesis).

**Table 5:** Number of valid and failed computations, number of non-computable *p* values, and percentage of discrepant inferential decisions with Holley and Guilford’s *G* (*n* = 64; 47,905 tables)

Estimator	Valid	Failed	no *p* value	*H*_1_ −	*H*_0_	*H*_1_ +	Total
SAC	47905	0	0	0.30	0.00	0.35	0.66
Gwet’s *AC*1	47905	0	2	5.49	3.98	0.00	9.46
Krippendorff’s *α*	47903	2	0	0.35	3.92	11.64	15.91
Scott’s *π*	47903	2	0	0.00	4.15	12.08	16.22
Pearson’s *r*	47649	256	0	7.23	6.60	7.23	21.07
Cohen’s *κ*	47903	2	254	7.21	6.70	7.21	21.12
Yule’s *Y*	47649	256	0	5.41	12.47	3.84	21.72
Yule’s *Q*	47649	256	0	8.38	5.30	8.38	22.06
Dice’s *F*1	47904	1	0	7.00	14.90	8.62	30.52
Shankar and Bangdiwala’s *B*	47903	2	0	0.00	18.25	16.45	34.70
Normalized McNemar’s *χ*^2^	47840	65	0	8.05	23.80	13.28	45.12
Traditional McNemar’s *χ*^2^	47840	65	0	9.78	21.15	18.72	49.65
Revised McNemar’s *χ*^2^ (2010)	47840	65	0	13.75	17.13	20.10	50.98
Revised McNemar’s *χ*^2^ (2017)	47903	2	0	13.82	16.69	23.70	54.21

### Comprehensive maps: all tables with 1 ≤ *n* ≤ 68

**Fig. 2:**
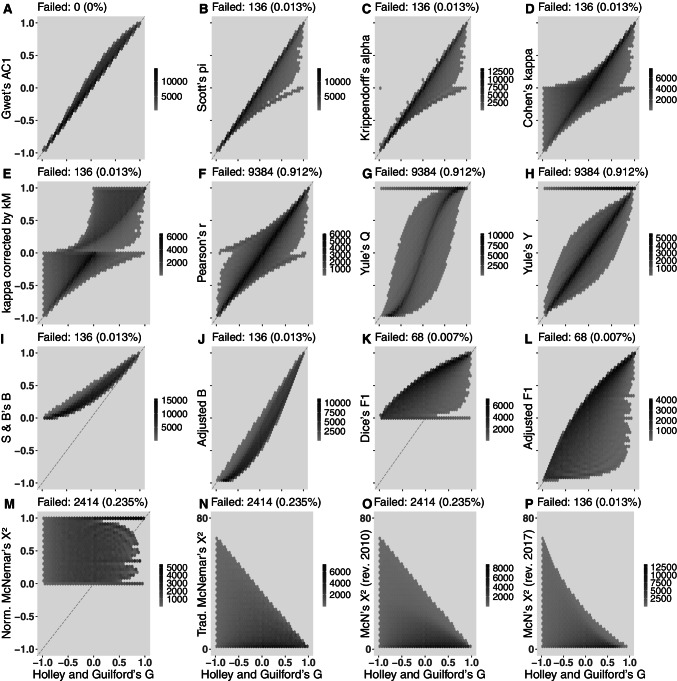
Hexbin plots of estimator computation of all possible 2x2 tables with *n* ranging from 1 to 68 (total of 1,028,789 tables). Failed is the count of non-computable tables. Holley and Guilford’s *G* was adopted as the benchmark. *Darker hexbins* correspond to the location of higher counts of tables

**Fig. 3:**
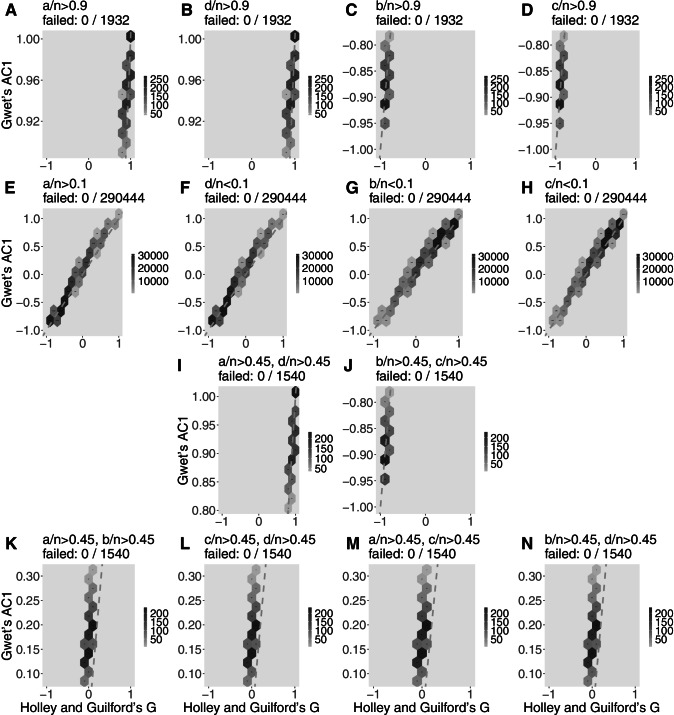
Hexbin plots of Gwet’s *AC*1 computation of extreme 2x2 tables with *n* ranging from 1 to 68 (total of 1,028,789 different tables; the percentage is of noncomputable tables) in comparison to Holley and Guilford’s *G* (adopted as the benchmark). First row (**A** to **D**): each cell contains more than 90% of all data. Second row (**E** to **H**): each cell contains less than 10% of all data. **I:** 90% of data in main diagonal; **J:** 90% of data in off-diagonal. Fourth row (**K** to **N**): respectively with 90% of data in the first row, second row, first column, and second column of a 2x2 table. The scale of the *y*-axis varies to show the pattern of coincidences between the estimators. The *dashed gray lines* represent the bisector

**Fig. 4:**
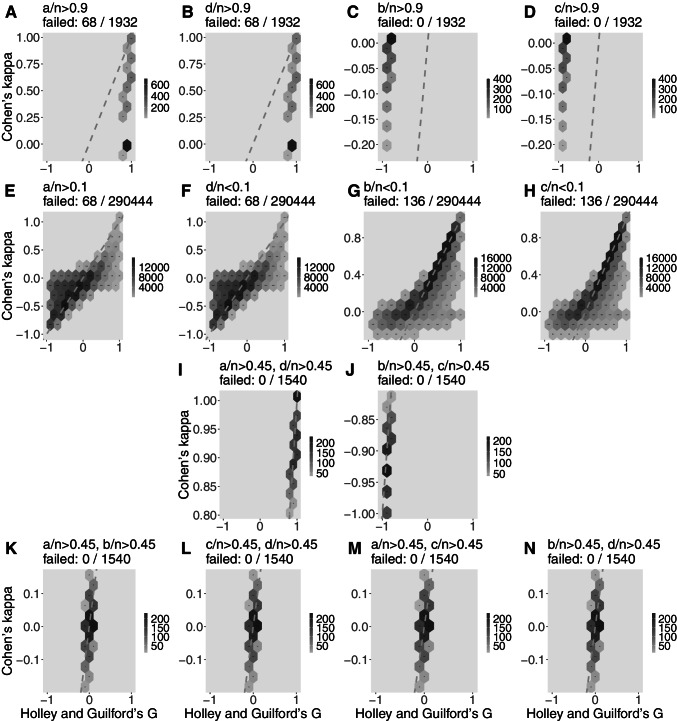
Hexbin plots of Cohen’s *κ* computation of extreme 2x2 tables with *n* ranging from 1 to 68 (total of 1,028,789 different tables; the percentage is of non-computable tables) in comparison to Holley and Guilford’s *G* (adopted as the benchmark). First row (**A** to **D**): each cell contains more than 90% of all data. Second row (**E** to **H**): each cell contains less than 10% of all data. **I:** 90% of data in main diagonal; **J:** 90% of data in off-diagonal. Fourth row (**K** to **N**): respectively with 90% of data in the first row, second row, first column, and second column of a 2x2 table. The scale of *y*-axis varies to show the pattern of coincidences between the estimators. The *dashed gray lines* represent the bisector

**Fig. 5:**
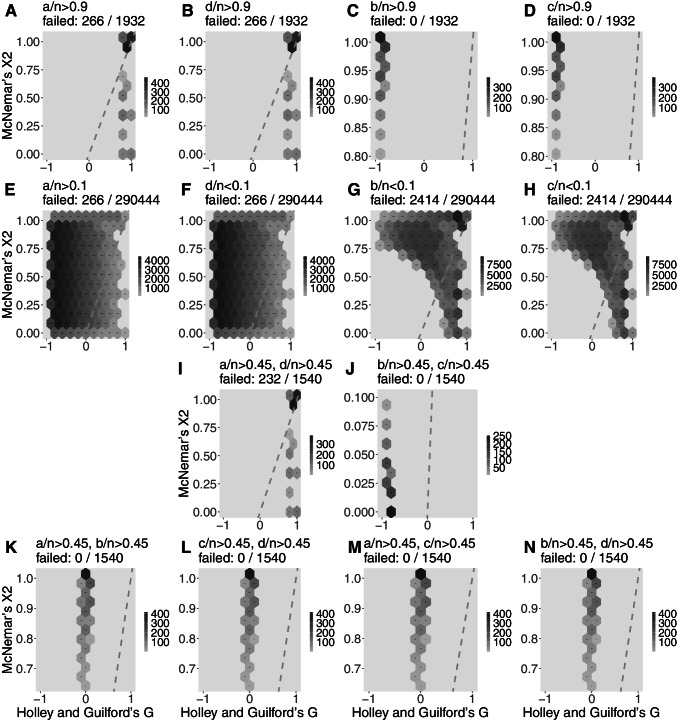
Hexbin plots of normalized McNemar’s *χ*^2^ computation of extreme 2x2 tables with *n* ranging from 1 to 68 (total of 1,028,789 different tables; the percentage is of non-computable tables) in comparison to Holley and Guilford’s *G* (adopted as the benchmark). First row (**A** to **D**): each cell contains more than 90% of all data. Second row (**E** to **H**): each cell contains less than 10% of all data. **I:** 90% of data in main diagonal; **J:** 90% of data in off-diagonal. Forth row (**K** to **N**): respectively with 90% of data in the first row, second row, first column, and second column of a 2x2 table. The scale of the *y*-axis varies to show the pattern of coincidences between the estimators. The *dashed gray lines* represent the bisector

Tables with size ranging from 1 to 68 observations were generated (see Supplemental Material “Creating all 2x2 tables with size *n*”), which resulted in 1,028,789 different 2x2 tables covering all possible arrangements of ‘*abcd*’. In addition to the results presented here, the stability of agreement coefficients were verified (not shown, see Supplemental Material “Computation of Figs. 2, 3, 4, and 5”).

Correlation between estimates of Holley and Guilford’s *G* (assumed as the benchmark) and all other studied estimators are ordered in Table 6.

**Table 6:** Pearson’s and Spearman’s correlations coefficients between Holley and Guilford’s *G* and other estimators. Table rows ordered by the median of Spearman’s correlations

	Pearson			Spearman		
Estimator	Median	HDI LB	HDI UB	Median	HDI LB	HDI UB
Gwet’s *AC*1	0.9931	0.9923	0.9934	0.9933	0.9899	0.9943
Shankar and Bangdiwala’s *B*	0.9698	0.9677	0.9713	0.9772	0.6699	0.9890
*B* rescaled to [-1,1]	0.9698	0.9677	0.9713	0.9772	0.6699	0.9890
Scott’s *π*	0.9555	0.9315	0.9643	0.9578	0.9386	0.9657
Krippendorff’s *α*	0.9555	0.9315	0.9643	0.9578	0.9392	0.9658
Pearson’s *r*	0.9131	0.9089	0.9474	0.8661	0.3033	0.9583
Cohen’s *κ*	0.8713	0.7973	0.8928	0.8659	0.7925	0.8897
Cohen’s *κ* corrected by *κ*_*M*_	0.8351	0.7770	0.8596	0.8371	0.7775	0.8604
Dice’s *F*1	0.7665	0.7349	0.7792	0.7611	0.7378	0.7751
*F*1 rescaled to [-1,1]	0.7665	0.7349	0.7792	0.7611	0.7378	0.7751
Yule’s *Q*	0.7841	0.7147	0.8326	0.7182	0.2305	0.8818
Yule’s *Y*	0.7384	0.6704	0.8000	0.7182	0.2305	0.8818
Normalized McNemar’s *χ*^2^	0.0968	0.0084	0.3324	0.1089	− 0.0316	0.6615
McNemar’s *χ*^2^ (rev. Lu, 2010)	− 0.3812	− 0.4037	− 0.2978	− 0.2991	− 0.3866	0.2988
Traditional McNemar’s *χ*^2^	− 0.3978	− 0.4202	− 0.3126	− 0.3066	− 0.3950	0.2880
McNemar’s *χ*^2^ (rev. Lu et al., 2017)	− 0.5299	− 0.5434	− 0.5289	− 0.5081	− 0.5301	− 0.2498

As defined in the previous section, Holley and Guilford’s *G* was adopted as the benchmark. It is possible to observe that Gwet’s *AC*1 has the best correlation with Holley and Guilford’s *G*, but many others also show acceptable correlations. However, the correlation was lower for Cohen’s *κ*, Yule’s *Q*, Dice’s *F*1, and Yule’s *Y*, and much lower, close to or absent, for normalized and traditional McNemar’s *χ*^2^.

A more detailed view of assessing the quality of each estimator is in Fig. 2. This is a representation in hexbin plots, an alternative to scatterplots when there is a large number of data points—in this case, a little more than one million points in each subfigure—in such a way that close-located points are overlapped into a single hexagonal bin (hence the name hexbin) and its color depends on the counts of collapsed points (the greater is the number of points, the darker is the hexbin). Each subfigure from A to L represents the point estimates obtained from Holley and Guilford’s *G* against all other estimators under assessment. The better the concordance between the estimators, the closer the darker hexbins can be to the bisector. According to this second criteria, again the best estimator is Gwet’s *AC*1 (Fig. 2A), and the worst is normalized McNemar’s *χ*^2^ (Fig. 2L). The traditional McNemar’s *χ*^2^ is not comparable to the other estimators because it does not provide values in the interval [-1,1] (although not shown here, its mapping was tested with procedures available in the supplemental material). Cohen’s *κ* (Fig. 2C), Pearson’s *r* (Fig. 2E), Yule’s *Y* (Fig. 2F) and *Q* (Fig. 2G), and Dice’s *F*1 (Fig. 2J) are mediocre estimators of agreement. Rescaled *F*1 aligned its darker hexbins with the bisector, but could not fix the number of tables with mistaken estimates below the bisector (Fig. 2K).

Shankar and Bangdiwala’s *B* (in its original form, Fig. 2H) is defective, with darker hexbins close to the bisector line only when *G* approaches 1. When scaled to the interval [-1,1] (Fig. 2I) the alignment to the bisector is improved but it leaves darker hexbins away and lighter hexbins close to the bisector line, meaning that it tends to underestimate in comparison to Holley and Guilford’s *G*. From Table 6, one should expect a better performance of *B*. In this figure, it is possible to observe that its excellent correlation depended on fairly aligned pairs of *G* and *B* observations but, in terms of linear regression, the great number of tables that are not close to the bisector leads to the not-so-good performance observed in Fig. 1. It is to say that such a simple rescaling of *B* cannot fix this estimator and it is structurally defective.

For more extreme 2x2 tables, Figs. 3, 4, and 5 show the pattern of Gwet’s *AC*1 (the estimator that better captured the estimates by *G*), Cohen’s *κ* (the most popular coefficient of agreement), and normalized McNemar’s *χ*^2^ (also popular but, again, the less reliable agreement estimator according to our analysis). In this analysis, Holley and Guilford’s *G* is again assumed as benchmark (*x* axis), while exemplary cases are detailed. The scales of the *y* axes are distorted, but dashed lines are represented to show the location of the bisector; in the same way as Fig. 2, the closer the darker hexbins are to the bisector, the better the estimator. Subfigures A to D show the pattern of the estimator when one of the contingency table cells (*a*, *b*, *c*, or *d*) concentrates 90% or more of all observations (therefore, A and B are cases of high agreement and C and D are cases of high disagreement). Subfigures E to H are the opposite scenarios: almost empty cells *a* to *d*, which provides a mixture of disagreement, randomness, and agreement cases—therefore, *G* may vary from -1 to 1 but it is expected that the other estimator should do the same along the bisector. Subfigures I and J are, respectively, concentration of observations in the main (agreement) and off (disagreement) diagonals. Finally, subfigures K to N are, respectively, the concentration of observations in the first row, second row, first column, and second column, which are situations that are mostly cases of neutrality.

Figure 3 reveals the good performance of Gwet’s *ACI* (attention to the values of *y* axes), in concordance with Holley and Guilford’s *G* in position and always presenting darker hexbins close to the bisector and some overestimation with values up to 0.3 in subfigures K to N, as expected from Fig. 2. Figure 4 analyzes Cohen’s *κ*, an intermediate estimator, showing discrepancies in subfigures A to D with hexbins away from the bisector and estimative around zero when should be computed agreement or disagreement. In the mixed situations illustrated in subfigures E to H, Cohen’s *κ* leaks around the bisector. In the other situations, its pattern is fairly adequate to the benchmark. Finally, Fig. 5 shows McNemar’s *χ*^2^ pattern providing estimates from 0 to 1 in subfigures A, B, and I (it uses only information from the off-diagonal), the reason for its better estimative in Figures C and D (although away the bisector because it provides only positive values), as well as in subfigure J, in which it computes values below 0.1 (here interpreted as consistent with disagreement between raters). In subfigures E to H, McNemar’s *χ*^2^ is erratic. In subfigures K to N, the pattern of this estimator provided values between 0.6 and 1.0, an overestimation of neutrality.

## Discussion

It was long understood by theoreticians that *G* is a superior estimator to Cohen’s *κ*, but practitioners of applied statistics, for some reason, adhere to the latter. Theoretical reasons and our findings led to the use of Holley and Guilford’s *G* index as a reference to the performance of concurrent estimators. More recently, Zhao et al. (2013) presented an extensive discussion on several agreement estimators, including situations with more than two observers or categories, and provided an important discussion on assumptions, frequently forgotten by applied researchers. It is beyond the scope of the present work, which is an application of several estimators to assess their performance, but it is interesting to emphasize that Gwet’s *AC*1 seems to provide fair estimates in uneven situations, which is arguable the most important and useful situations for researchers.

In fact, many authors compare several of the estimators but, to our knowledge, no one performed an exhaustive analysis with hundreds of thousands of tables as presented here to obtain a comprehensive map of estimator patterns. Many comparisons stick to some particular cases, using challenging tables similar to that in Tables 3 and 4, sometimes to show the weakness or strength of particular coefficients in particular situations. Even so, many of their conclusions point to many of Cohen’s *κ* problems and favor Holley and Guilford’s *G* (Shreiner, 1980; Green, 1981) or Gwet’s *AC*1 (Kuppens, Holden, Barker, & Rosenberg, 2011; Wongpakaran et al., 2013; Xie et al., 2017).

Accordingly with our results, by assuming Holley and Guilford’s *G* as the benchmark, Gwet’s *AC*1 is also a good estimator. Not only *AC*1 mistakes are the lowest in inferential statistics, but also these mistakes are located (Fig. 1C) around the null hypothesis, eventually assuming low agreement when there is none, and in the low disagreement area, eventually failing in detecting it, which is—paraphrasing Cohen (1960)—“unlikely in practice”. In the most important area of agreement between raters (*H*_1_ + in Fig. 1C), *AC*1 was the only coefficient with no mistaken inferential decisions (Table 5). *AC*1 agreement is also close to the bisector line (darker hexbins in Fig. 2A) and it is not confounded by extreme tables (Fig. 3). In a recent study, Konstantinidis, Le, & Gao (2022) compared the performance of Cohen’s *κ*, Fleiss’ *κ*, Conger’s *κ*, and Gwet’s *AC*1, with the difference that the authors had to apply simulations because of the explosive number of possible tables with greater samples, up to 500 observations, exploring tables with multiple observers, thus fixing the agreement to known values to compare the relative performance of these estimators. Conger’s *κ* (1980) was not approached in our study for it is a generalization for more than two observers and, therefore, it does not apply to 2x2 tables. The main conclusion of these authors is in agreement with our results, being Gwet’s *AC*1 superior to the other statistics, especially with what we called more extreme tables (these authors named it asymmetric cases).

Cohen’s *κ* is not only a poor estimator of agreement but also is prone to provide incorrect statistical decisions when there is neutrality and no large disagreement or agreement between raters. Fig. 1G and Table 5 show that it is mistaken in around 21% of all possible tables with *n* = 64, with roughly one-third in each region, but this distribution is not uniform: mistakes are less likely to occur away from the greater disagreements or agreements: the solid line of Cohen’s *κ* has two peaks in the transition from the gray area (null hypothesis) to the white areas (rejection of *H*_0_), thus it is when there is low disagreement or agreement that Cohen’s *κ* is more subjected to inferential errors. This weakness is not visible in the low agreement or disagreement scenarios in Table 3, which denotes the importance of exhaustive computation of many tables; to draw general conclusions from the study of particular cases may be misleading. Another weakness is that there were problems in 256 tables of size *n* = 64 (Table 5). Two tables failed when all data are in *a* or *d* (a clear 100% agreement) because it causes a division by zero (equation 3). In addition to that, the inferential decision provided by epiR::epi.kappa (*z* statistic for *κ* associated with *p* value) is also unavailable in other 254 tables when one row or column is empty (i.e., *a* + *b* = 0 or *a* + *c* = 0 or *b* + *d* = 0 or *c* + *d* = 0). Finally, Fig. 4 shows some details of *κ* limitations. In scenarios of agreement in which most of data are concentrated in *a* or *d*, *κ* mistakenly provide values close to 0 (the darkest hexbins in Fig. 4A and B). That happens due to Eq. 3, for *a* and *d* appear in both parcels of the denominator creating an exaggerated value while the parcel *ad* in the numerator is a low value, thus *κ* underestimates the agreement in these situations. Concentration in *b* or *c*, which appears only one in each denominator parcel, only causes a small number in the numerator due to *bc* (always a small number due to high *b* and low *c* or vice-versa), leading to underestimation of the disagreement (Fig. 4C and D). Cohen’s *κ* also has problems when only one of the four cells is relatively empty, which may happen, for instance, if one of the raters is more lenient than the other; in these cases, Cohen’s *κ* assessment has an excess of variability (Fig. 4E to H). The last comment on Cohen’s *κ* is that the correction by maximum *κ*, proposed by Cohen himself and forgotten by following researchers, may be a correction for effect size (Table 2) but it worsened the general estimator pattern (compare Fig. 2D and C).

We started this review by criticizing Cohen’s *κ* but the investigation of alternatives led, in the end, to the development of a method to assess any other estimator, some of them described in this text. For that, McNemar’s *χ*^2^ was collateral damage. It was not anticipated such poor performance for such a widespread estimator. On the other hand, we tested the performance of McNemar’s *χ*^2^ to verify if it could be applied to a general situation of agreement, concluding that it cannot be used. To be fair, one must recognize that McNemar’s *χ*^2^ test was designed for a very specific situation: change of signal in a pre-post scenario, using only the off-diagonal (McNemar, 1947). Unfortunately, McNemar’s *χ*^2^ sometimes is applied in the context of verifying agreement between methods (e.g., Kirkwood and Sterne, pp. 216-218, 2003). By using only *b* and *c*, it becomes a futile mental exercise to link rejection or non-rejection of the null hypothesis with agreement or disagreement between raters. For instance, in trying to interpret McNemar’s measure as agreement between raters, *b* = 26,*c* = 10 leads to *MN* = 0.44,*CI*95*%*(*MN*) = [0.14,0.74], rejects *H*_0_ and would suggest disagreement, but *b* = 18,*c* = 18 (which is the same amount of disagreement) leads to *MN* = 0.00,*CI*95*%*(*MN*) = [− 0.02,0.34], does not reject *H*_0_ and would suggest agreement. Both decisions completely disregard the agreement values, *a* and *d*, it does not matter if they are 2 or 2 thousand. It would only matter that one rater systematically opposes the other and that one of them is biased to provide much more positives or negatives than the other. If they are perfectly opposed to making *b* = *c*, then this perfect disagreement would be no more detectable. Revised versions designed to include more table information by adding *a* and *d* to the play do not seem to improve this test’s ability to measure agreement. Consequently, the application of McNemar’s *χ*^2^ as a measure of association or agreement, when removed from its original context, can only lead to the confusing results observed on the challenge 2x2 tables with the inability to detect agreement or disagreement in some situations and overestimation of agreement or disagreement in others (Tables 3 and 4), fails in non-rejection of the null hypothesis (Fig. 1L to O), and mixed estimates along clear situations of agreement or disagreement (Fig. 2M to P). In essence, McNemar’s *χ*^2^ is not an agreement estimator and its use must be restricted to its original context.

To close this discussion, a brief comment on the other estimators is in order. Scott’s *π* appears in third place. It is not a bad estimator but, contrary to its original proposition as inter-rater reliability, it seems a more reliable estimator of disagreement (Fig. 1E). Another way to confirm this statement is to observe that most of its correct counts are on the bisector, but the dispersion increases with increasing rater agreement (Fig. 2B). Krippendorff’s *α* (Fig. 2C) showed pattern similar to Scott’s *π*, as predicted by theory for this case of nominal variables and two raters. Perhaps, being Krippendorff’s *α* a generalization of other coefficients, its pattern depends on the dimensions and nature of the involved variables, inheriting the strengths and weaknesses of the respective coefficients that it may emulate.

Pearson’s *r* is, primarily, a measure of association. However, in 2x2 tables its performance is also very similar to Cohen’s *κ*, conceived to measure agreement, which can be observed by similar density plots (Fig. 1F and G), mapping of hexbins (Fig. 2F and D) or their similar correlations with *G* in Table 6. We observe that equations for *κ* (Eq. 3), *Q* (Eq. 12), *r* (Eq. 23, which is also equal to *ρ*, *τ*, *ϕ*, and Cramér’s *V*), all have a sort of *OR* (Eq. 13) in their numerators (*ad* − *bc*). Although having similar global performances, the deficiencies of *r* and *κ* are due to different reasons (see Tables 3 and 4).

Yule’s *Q* and *Y* come next, with small advantage to *Y*. Their global performance are close to that of Cohen’s *κ* and Pearson’s *r*, but *Y* made more mistakes when there is neutrality (i.e., in the region of the non-rejection of the null hypothesis), while *Q* made relatively more mistakes when there are disagreement or agreement between raters (i.e., in the regions of rejection of the null hypothesis, Fig. 1H and I). Both also show a tendency to overestimate agreement providing values equal to 1 for any disagreement, neutrality or agreement provided by *G*, which is represented by the horizontal lines on the top of Figs. 2G and 2H, specially for higher agreements (darker hexbins); this also explains the results observed in Table 4. At least, as it happens to be with Scott’s *π* (Fig. 2B), Yule’s *Y* concentrates most of their estimates around the bisector line, while Yule’s *Q* is more problematic, showing a sigmoid shadow of darker hexbins (Fig. 2G) that explains its greater tendency do overestimate both agreement and disagreement as shown in Table 3.

Dice’s *F*1 and Shankar and Bangdiwala’s *B* only provide positive estimates in their original forms. Since it is confusing to compare a range [0,1] with [-1,1] provided by other coefficients, we proposed the rescaling by 2*i* − 1 (where *i* is *F*1 or *B*). This is a linear transformation that does not affect these coefficient properties, with zero now corresponding to neutrality or randomness, negative values to disagreement, and positive values to agreement between raters. The graphical effect of this transformation is to approach the pattern of computed coefficients to the bisector (Figure 2K to L and Figure 2I to J). Rescaled Dice’s *F*1 (Fig. 2L) fills around half of the graph area, which indicates an inability to detect many occurrences of agreement between raters. Incidentally, *F*1 does not use the entire information from a 2x2 table, leaving *d* out of reach (the Traditional McNemar’s *χ*^2^ that showed poor performance also uses incomplete information). Perhaps, the first criteria to be a good agreement estimator should be to apply the whole information available.

Shankar and Bangdiwala’s *B* has other type of serious problems. Except for the McNemar’s versions, it was the estimator that more mistakenly rejected the null hypothesis in situations of neutrality and failed to reject the null hypothesis in agreement situations, *H*_1_ + (Fig. 1K and Table 5). Like Scott’s *π* (Fig. 1E), it has no mistakes on the H1- area—although the performance of *π* is a lot better.

In a nutshell, the estimators can be divided, according to section “Association and agreement”, in measurements of association (Eq. 36) or agreement (Eq. 37), depending mainly on the structure of the numerator of their respective equations. It is shown in Table 7 that most of the estimators usually taken as a measurement of agreement are merely estimates of association; only two of the estimators analyzed in this work are authentic agreement coefficients.

**Table 7:** Nature of estimators

Estimator	Numerator	Classification
Holley and Guilford’s *G*	(*a* + *d*) − (*b* + *c*)	Agreement
Gwet’s *AC*1	$\left (a^{2}+d^{2}\right ) - \frac {(b+c)^{2}}{2}$	Agreement
Adjusted Dice’s *F*1	2*a* − (*b* + *c*)	Mostly agreement
Cohen’s *κ*	2(*ad* − *bc*)	Association
Yule’s *Q*	*ad* − *bc*	Association
Yule’s *Y*	$\sqrt {ad} - \sqrt {bc}$	Association
Odds ratio	$\frac {ad}{bc}$ or *log*(*ad*) − *log*(*bc*)	Association
Pearson’s *r*	*ad* − *bc*	Association
Scott’s *π*	$ad - \left (\frac {b+c}{2} \right )^{2}$	Mixed
Rescaled Shankar and Bangdiwala’s *B*	$\left (a^{2}+d^{2}\right ) - (2bc + (a+d)(b+c))$	Mixed
McNemar’s *χ*^2^	$\frac {b}{c}$ or *log*(*b*) − *log*(*c*)	Undefined

This work has a humble mission for restoring Holley and Guilford’s *G* as the best agreement estimator, closely followed by Gwet’s *AC*1. Both have inferential statistics associated with them, to satisfy research requirements. Gwet’s *AC*1 was already implemented in R packages. We could not find any Holley and Guilford’s *G* implementation but the R scripts presented in the Supplemental Material in the Section named “Implementation of Holley and Guilford’s *G*” can be easily adapted, including the asymptotic test proposed in the literature for tables with *n* > 30 (bootstrapping techniques are easy to adapt for smaller tables). Holley and Guilford’s *G* and Gwet’s *AC*1 should be considered by modern researchers as the first choices for agreement measurement in 2x2 tables.

### Supplementary Information

ESM 1 (PDF 530 KB)
