# Supplementary material for: Better to be in agreement than in bad company: A critical analysis of many kappa-like tests
Source: Behav Res Methods. 2022 Sep 16;55(7):3326–47. doi: 10.3758/s13428-022-01950-0 (PMC10615996; doi:10.3758/s13428-022-01950-0)
Supplement: Supplementary file 1 — (PDF 530 KB) [file 13428_2022_1950_MOESM1_ESM.pdf]

# Supplemental Material

related to:

Paulo Sergio Panse Silveira, Jose Oliveira Siqueira (2022)
Better to be in agreement than in bad company: a critical analysis of many kappa-like tests.
*Behavior Research Methods* https://doi.org/10.3758/s13428-022-01950-0

All procedures and data applied along this supplemental material were implemented in R and are available to download from Harvard Dataverse

https://doi.org/10.7910/DVN/HMYTCK

The following sections are:

- ("R functions") describes the implementation of all mentioned estimators, including their inferential statistic tests.
- ("Computation of Tables 3 and 4") replicates findings from Tables 3 and 4.
- ("Creating all 2x2 tables with size $n$") has the procedures to generate all possible tables, given any range of $n$.
- ("Computation of Figure 1 and Table 5") exemplifies the creation of all 47,905 possible tables with $n = 64$ to build Figure 1.
- ("Computation of Table 6") shows the procedure to replicate Table 6 and some additional figures.
- ("Computation of Figures 2, 3, 4, and 5") shows how to create 1,028,789 possible tables with $1 \leq n \leq 68$ and provides the R code to generate hexbin Figures 2, 3, 4, and 5.

Estimators were computed according the following convention:

| | $B$ | $\bar{B}$ | |
|---|---|---|---|
| $A$ | $a$ | $b$ | $a+b$ |
| $\bar{A}$ | $c$ | $d$ | $c+d$ |
| | $a+c$ | $b+d$ | $a+b+c+d$ |

In this table, $A$ and $B$ are representations of events (positive observer evaluation, existence of disease, exposition or effect, etc.) and $\bar{A}$ and $\bar{B}$ are their respective negations (negative observation, health individuals, absence of exposition or effect, etc.). In the main diagonal, $a$ and $d$ are counting or proportions of positive and negative agreements. In the off-diagonal, $b$ and $c$ are counting or proportions of disagreements. Sample size along this text is $n = a + b + c + d$.

Implementations applying functions from several R packages were also incorporated in our experiments for two main reasons:

1. to check if the point-estimate using the $abcd$-based formulas were correct, and
2. to take advantage of inferential statistics already implemented in R packages.

In some cases we could not locate any implementation in R packages, thus we applied bootstrapping to obtain, at least, a binary decision (rejection or non-rejection of the null hypothesis) based on confidence interval 95%. Details are described in this appendix, section "**Implementation of Holley and Guilford's *G***".

In order to show the application of the following procedures, we adopted an example from the results of Bell and Kato-Katz examination performed on each of 315 stool specimens [16]. `Data` is a contingency table that can be incorporated in matrix `m` with:

```
Data <- ("
  BellxKK P   N
  P       184 54
  N       14  63
")
```

```
m <- as.matrix(read.table(textConnection(Data),
                          header=TRUE, row.names=1))
print(m)
```

Sometimes, according to the required input by some functions, an extensive presentation in data frame, `dt`, can be obtained with:

```
dt <- DescTools::Untable(m)
print(dt)
```

It follows the implementation of all estimator functions (including the indications of filenames in which these functions can be found) applied for the current work.

## R functions

### Implementation of Cohen's kappa ($\kappa$) and corrected kappa ($\kappa_M$)

The computation of Cohen's $\kappa$ [3] and his proposed correction by maximum $\kappa$ were implemented by two functions included in the file `agr2x2_kCohen.R`:

- The function `agr2x2_kCohen` receives the parameters `a`, `b`, `c`, and `d`, or, alternatively, the matrix `m` in place of the first parameter (in this case, internally converting the matrix in `a`, `b`, `c`, `d`), to implement equation 3; it returns a matrix containing the essential computation: $\kappa$, $\kappa_M$ and $p$.
- The auxiliary function `agr2x2_maximum_kCohen` computes the maximum kappa ($\kappa_M$, equation 4). It is called from `agr2x2_kCohen` only when $\kappa > 0$.

The inferential statistics is provided by `epiR::epi.kappa`, executed only if `test=TRUE` to capture its $p$ value. Since Cohen's $\kappa$ is a measure of agreement between observations, higher values of $\kappa$ lead to rejection of the null hypothesis ($H_0 : \kappa = 0$), thus providing evidence for agreement between observers or methods (generically named as raters along this text) when $\kappa$ is positive. Conversely, significantly negative values of $\kappa$ suggests disagreement between raters.

Cohen's$\kappa$using the Bell and Kato-Katz's example is computed by:

```
source("agr2x2_kCohen_example.R")
```

The output is:

```
    P  N
P 184 54
N  14 63

H0: kappa = 0
        k        kM           p
 0.502924 0.7076023 1.020088e-20
```

### Implementation of Holley and Guilford's $G$

The computation of Holley and Guilford's $G$ (equation 6) and the inferential asymptotic statistical test (equation 11) proposed by Lienert [30] were implemented in function `agr2x2_G` (available in file `agr2x2_G.R`) according to section "Methods, Holley and Guilford's $G$" in the main text.

Holley and Guilford's $G$ using the Bell and Kato-Katz's example is computed by:

```
source("agr2x2_G_example.R")
```

The output is:

```
    P  N
P 184 54
N  14 63

H0: G = 0
        G p
 0.568254 0
```

### *Bootstrapping*

Equation 11 is an asymptotic approach, thus it is formally applied to $n \geq 30$. It is to say that it may or may not work for smaller tables, which is the case in the present work for we studied the range from $n = 1$ to $n = 68$. For that, it was implemented another version (called Simple Agreement Coefficient, $SAC$) with inferential statistical decision by bootstrapping, which is a robust statistical method based on resampling with replacements, independent of sample size and of variable distribution [8]. Inferential decision by bootstrapping depends on confidence interval, thus the rejection or non rejection of the null hypothesis if binary (see the details for the binary version of $p$ value described under "**Implementation of Scott's** $\pi$" in this appendix).

A bootstrapping is performed with thousands of repetitions (`B` resamplings), calling `agr2x2_boot.table.R` to generate variant 2x2 tables based on the table under investigation and computing a value of $SAC$ for each one. The prediction interval is provided by the high density interval of the distribution of these $SAC$ values (using `HDInterval::hdi`). The non-rejection of this null hypothesis (`pbin=1`) occurs when zero is inside the prediction band (it implies randomness between raters), while its rejection (`pbin=0`) is interpreted as non-null $SAC$ (which is evidence of agreement when $SAC > 0$ or disagreement when $SAC < 0$ between raters).

$SAC$ using the Bell and Kato-Katz's example is computed by:

```
source("agr2x2_SAC_example.R")
```

The output shows the point-estimate of $SAC$, the Lower and Upper Band of the prediction band of 95%, the $p$ value (in this example, rejecting $H_0$ because zero is not contained in the interval [SACLB,SACUB], and the time spent to compute this bootstrapping:

```
    P  N
P 184 54
N  14 63

H0: SAC = 0
100000 resamples:
      SAC     SACLB     SACUB pbin
 0.568254 0.4764835 0.6575709    0
Time difference of 1.545686 mins
```

### Implementation of Yule's $Q$

The function `agr2x2_qYule` implements equation 12. When descriptive statistics is requested, it applies `exact2x2::fisher.exact`.

The null hypothesis of Yule's $Q$ is the populational independence between raters. The rejection of the null hypothesis is interpreted as evidence of agreement ($Q > 0$) or disagreement ($Q < 0$) between raters:

Yule's $Q$ using the Bell and Kato-Katz's example is computed by:

```
source("agr2x2_qYule_example.R")
```

The output is:

```
    P  N
P 184 54
N  14 63

H0: Q = 0
        Q     p.value
 0.877551 1.49462e-20
```

### Implementation of Yule's $Y$

Similar to Yule's $Q$, this estimator was implemented by `agr2x2_yYule` (equation 18). Since we did not locate any implementation of this estimator nor any suitable inferential statistical test, it was implemented by bootstrapping (it also depends on `agr2x2_boot.table`, which is described in " **Implementation of Holley and Guilford's $G$**" - see $SAC$).

Yule's $Y$ using the Bell and Kato-Katz's example is computed by:

```
source("agr2x2_qYule_example.R")
```

The output is analogous to that of $SAC$:

```
    P  N
P 184 54
N  14 63

H0: Y = 0

100000 resamples:
        Y      Y.LB     Y.UB pbin
 0.593147 0.4880959 0.704123    0
Time difference of 1.541523 mins
```

### Implementation of Pearson's $r$

The function `agr2x2_rPearson` implements equation 23. When descriptive statistics is requested, it applies the function `cor.test`. Observe that `cor.test` requires two numeric vectors to compute and test Pearson's correlation, thus it has to be preceded by `DescTools::Untable`.

The null hypothesis is absence of populational correlation. The rejection of this null hypothesis is interpreted as evidence of agreement ($r > 0$) or disagreement ($r < 0$) between raters.

Pearson's $r$ using the Bell and Kato-Katz's example is computed by:

```
source("agr2x2_rPearson_example.R")
```

The output is:

```
    P  N
P 184 54
N  14 63

H0: r = 0
         r     p.value
 0.5259078 8.39619e-24
```

## Implementation of McNemar's $\chi^2$

The function agr2x2_mnMcNemar implements equation 25, which corresponds to the Normalized version of McNemar's $\chi^2$. When descriptive statistics is requested, it applies bootstrapping (described in " **Implementation of Holley and Guilford's** $G$" - see $SAC$).

Unlike other tests, the null hypothesis of McNemar's test is a test of change, therefore the rejection of the null hypothesis is taken as change and not necessarily evidence of agreement or disagreement between raters (see section "**Discussion**" in the main text). It also ranges from 0 to 1, in such a way that 0 corresponds to randomness and 1 to the maximum of change.

Normalized McNemar's $\chi^2$ using the Bell and Kato-Katz's example is computed by:

```
source("agr2x2_mnMcNemar_example.R")
```

The output is:

```
    P  N
P 184 54
N  14 63

H0: MN = 0
100000 resamples:
        MN     MN.LB     MN.UB pbin
 0.5882353 0.3932159 0.7807927    0
Time difference of 1.662342 mins
```

Although not analyzed along the main text because it does not return values in the interval [-1,1], the traditional McNemar's $\chi^2$ was implemented in

– agr2x2_chi2mnMcNemar

When the inferential test is required, it is performed by exact2x2::mcnemar.exact computing the estimate of probability-ratio and confidence interval given by $\frac{b}{c}$; the null hypothesis is rejected when the unitary value is not included in its confidence interval 95%.

Other attempts to improve this estimator [31,32] were also implemented using bootstrapping (there is no function for these variations available in R), respectively, in:

– agr2x2_mnMcNemar2010 and
– agr2x2_mnMcNemar2017

These three variations of McNemar's $\chi^2$ using the Bell and Kato-Katz's example can be computed by:

```
source("agr2x2_chi2mnMcNemar_example.R")
```

The output is:

```
    P  N
P 184 54
N  14 63

Traditional McNemar's X2:
H0: MN = 0
   Chi2MN      b/c   b/c.LB   b/c.UB            p
 23.52941 3.857143 2.112958 7.519343 1.109726e-06

Modification of Lu (2010)
 DOI 10.1080/03610920903289218:
H0: MN(2010) = 0
100000 resamples:
   MN2010 MN2010LB MN2010UB pbin2010
 13.40283 5.086326 22.78276        0
Time difference of 1.708656 mins
```

```
Modification of Lu et al. (2017)
 DOI 10.1080/03610926.2016.1228962:
H0: MN(2017) = 0
100000 resamples:
   MN2017 MN2017LB MN2017UB pbin2017
 5.958574 1.865308 10.90186        0
Time difference of 1.688534 mins
```

All versions of McNemar's $\chi^2$ only provide positive values computed from the off-diagonal, thus they cannot distinguish situations of agreement of disagreement between raters.

### Implementation of Scott's $\pi$

The function `agr2x2_piScott` implements equation 30.

When descriptive statistics is requested, it applies the function `rel::spi` (which requires `DescTools::Untable`; see "**Implementation of Pearson's $r$**"). Unfortunately, `rel::spi` provides only a confidence interval 95% of $\pi$ without $p$ value. The null hypothesis is $H_0 : \pi = 0$, i.e., absence of agreement or disagreement.

Lacking a $p$ value, our proposition is a binary decision, a "binary $p$ value", assuming the non-rejection of this null hypothesis when `pbin=1`, implying randomness between raters (neutrality). Since Scott's $\pi$ is an index of agreement between raters, when the null hypothesis is rejected (`pbin=0`), it implies that the higher the absolute value the greater is the agreement (if $\pi > 0$) or the disagreement (if $\pi < 0$) between raters.

Scott's $\pi$ using the Bell and Kato-Katz's example is computed by:

```
source("agr2x2_piScott_example.R")
```

The output is:

```
    P  N
P 184 54
N  14 63
            pi      piLB      piUB p_bin
[1,] 0.4935212 0.3863408 0.6007017     0
```

### Implementation of Krippendorff's $\alpha$

This coefficient is implemented with a new R package developed by John Hughes [20], including inferential decision by bootstrapping which is called from our implementation when `B` is numeric (this implementation requires a numeric data frame to process, using `DescTools::Untable` and some additional transformations).

Krippendorff's $\alpha$ using the Bell and Kato-Katz's example is computed by:

```
source("agr2x2_alphaKrippendorff_example.R")
```

The output is:

```
    P  N
P 184 54
N  14 63

H0: alpha = 0
100000 resamples:
     alpha   alphaLB   alphaUB pbin
 0.4943252 0.3827792 0.5984347    0
Time difference of 1.570461 mins
```

Theory states that Krippendorff's $\alpha$ coincides asymptotically with Scott's $\pi$ for nominal variables with two raters [43]. This is testable with the current methods along 2x2 tables of sizes ranging $1 \leq n \leq 68$ and the result is the following figures:

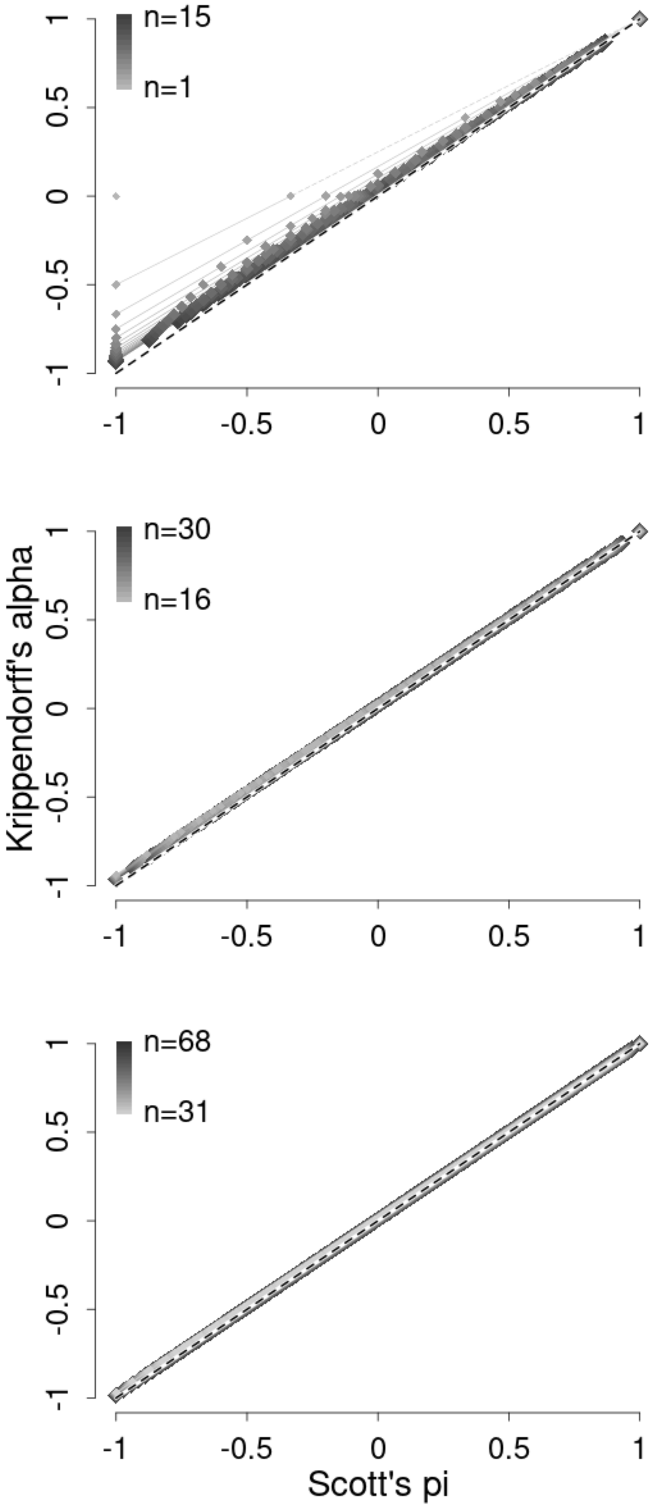


Comparison of point-estimates of Scott's $\pi$ and Krippendorff's $\alpha$. Dashed line is bisector: observe bias of Krippendorff's $\alpha$ in smaller table sizes, greater with increasing disagreement between raters. For table size $ge$ 30, estimatives are close to the bisector, thus confirming the asymptotic pattern expected by theory.

**Implementation of Dice's $F1$ and Adjusted $F1_{adj}$**

Dice's $F1$ is a measure of agreement providing only positive numbers (ranging from 0 to 1), it is assumed that the higher the value of $F1$, the greater is the agreement between raters (0 is disagreement and 0.5 is randomness). Consequently, we propose to rescale $F1$ (Equation 31) to $F1_{adj}$ (Equation 32).

To our knowledge, there is no inferential test implemented to date in R packages. Therefore, we circumvent this problem with bootstrapping (see bootstrapping on "**Implementation of Holley and Guilford's** $G$" for details).

Equations 31 and 32 and inferential tests were implemented in `agr2x2_f1Dice.R`.

Dice's $F1$ using the Bell and Kato-Katz's example is computed by:

```
source("agr2x2_f1Dice_example.R")
```

The output is:

```
    P  N
P 184 54
N  14 63

H0(F1): F1 = 0.5
H0(F1.adh): F1 = 0
bootstrapping
100000 resamplings
                     F1     F1.LB     F1.UB pbin
F1 (regular)  0.8440367 0.8065910 0.8799592    0
F1 (rescaled) 0.6880734 0.6131821 0.7599185    0
Time difference of 3.558388 mins
```

**Implementation of Shankar and Bangdiwala's $B$**

This coefficient provides only positive numbers (ranging from 0 to 1) and equation 33 was implemented in `agr2x2_bShankar.R`. It is assumed that the higher the value of $B$, the greater is the agreement between raters (0 is disagreement and 0.5 is neutrality). The inferential statistical decision depends on bootstrapping (see bootstrapping on "**Implementation of Holley and Guilford's** $G$" for details).

Shankar and Banddiwala's $B$ using the Bell and Kato-Katz's example is computed by:

```
source("agr2x2_bShankar_example.R")
```

The output is:

```
    P  N
P 184 54
N  14 63

H0: B = 0.5
100000 resamples:
           SB     SB_LB     SB_UB pbin
[1,] 0.673846 0.6079652 0.7410193    0
Time difference of 1.674844 mins
```

Our proposition for rescaling $B$ (equation 34) is similar to the procedure of "**Implementation of Dice's** $F1$ **and Adjusted** $F1_{adj}$". It was implemented in `agr2x2_badjShankar.R`.

Rescaled Shankar and Banddiwala's $B$ using the Bell and Kato-Katz's example is computed by:

```
source("agr2x2_badjShankar_example.R")
```

The output is:

```
    P  N
P 184 54
N  14 63

H0: B = 0
100000 resamples:
         SBadj  SBadj_LB  SBadj_UB pbin
[1,] 0.3476921 0.2152926 0.4813354    0
Time difference of 1.547922 mins
```

### Implementation of Gwet's $AC1$

The function agr2x2_ac1Gwet implements equation 35, receiving the same parameters and returning a matrix alike the function agr2x2_kCohen (see "**Implementation of Cohen's kappa ($\kappa$) and corrected kappa ($\kappa_M$)**").

Gwet's $AC1$ is an index of agreement between observations, thus the higher the absolute value the greater is the agreement ($AC1 > 0$) or disagreement ($AC1 < 0$) between raters.

One advantage of Gwet's $AC1$ is her proposal of an estimator ballasted on a statistical test with a defined sample distribution, which leads to the computation of a $p$ value. This inferential statistics is implemented in R by irrCAC::gwet.ac1.raw, which is called from our implementation when test=TRUE (this function requires a numeric data frame to process, using DescTools::Untable and some additional transformations).

Gwet's $AC1$ using the Bell and Kato-Katz's example is computed by:

```
source("agr2x2_ac1Gwet_example.R")
```

The output is:

```
    P  N
P 184 54
N  14 63

H0: AC1 = 0
       AC1 p
 0.6237683 0
```

### Comment on null hypotheses

McNemar's $\chi^2$ testing is the only estimator whose null hypothesis is reverse, concluding for absence of change (i.e., $b = c$) when the $p$ value is significant (all other proposed estimators conclude for agreement or disagreement by rejection of their respective null hypotheses).

For the Bell and Kato-Katz example applied by Kirkwood and Sterne, 2003, pp. 216-218 [24], McNemar's $\chi^2$ leads to rejection of the null hypothesis and the authors concluded for the disagreement between methods, stating superiority of @@. This is diverse from the other concurrent estimators applied in the current work suggesting that both methods are equivalent (without any judgment of superiority). The problem is that the misuse of McNemar's $\chi^2$ that cannot be applied for agreement/disagreement decisions, as discussed in the main text.

It order to further emphasize that McNemar's $\chi^2$ is not an agreement estimator we apply all implemented functions to a hypothetical matrix that show obvious agreement between two raters scoring a hypothetical measurement method as positive or negative,

Using the Bell and Kato-Katz's example with:

```
source("agr2x2_4tests.R")
```

The output is:

```
         Positive Negative
Positive       70        2
Negative        4       40

---- Cohen's kappa ----
        k        kM           p
 0.889172 0.9630573 9.406842e-22

---- Yule's Q ----
        Q      p.value
 0.994302 4.765713e-24

---- Pearson's r ----
         r      p.value
 0.8897794 1.220942e-40

---- Scott's pi ----
        pi      piLB     piUB p_bin
 0.8891367 0.8014564 0.976817     0

---- Krippendorff's alpha ----
     alpha   alphaLB   alphaUB pbin
 0.8896145 0.7976266 0.9632048    0

---- Dice's F1 ----
                      F1     F1.LB     F1.UB pbin
F1 (regular)  0.9589041 0.9233024 0.9898247    0
F1 (rescaled) 0.9178082 0.8466048 0.9796493    0

---- Gwet's AC1 ----
       AC1 p
 0.9030371 0

---- SAC ----
       SAC     SACLB     SACUB pbin
 0.8965517 0.8078143 0.8199408    0

---- Holley and Guilford's GC ----
         G p
 0.8965517 0

---- Normalized McNemar's chi-squared ----
        MN       MN.LB     MN.UB pbin
 0.3333333 -0.06005009 0.9364187    1

---- Traditional McNemar's chi-squared ----
    Chi2MN b/c     b/c.LB   b/c.UB      p
 0.6666667 0.5 0.04522901 3.488772 0.6875

---- Rev. Lu (2010) McNemar's chi-squared ----
    MN2010   MN2010LB MN2010UB pbin2010
 0.4113475 -0.2927907 3.315413        1

---- Rev. Lu et al. (2017) McNemar's chi-squared ----
     MN2017    MN2017LB MN2017UB pbin2017
 0.03695444 -0.03145867 0.344082        1
```

Observe the similarity of the point-estimate of all estimators and the rejection of null hypothesis. All variants of McNemar's $\chi^2$ are discrepant regarding both estimated value and $p$ value. A McNemar's test does not measure agreement, but tests $H_0 : b = c$, which is applicable to pre-post situations. For instance, after and before an intervention (for instance, a electoral debate) it can be applied to verify changes of opinions from '-' to '+' ($b$) or '+' to '-' ($c$) of a group of voters.

### Computation of Tables 3 and 4

In order to create challenge scenarios, two worksheets (Excel format) with 2x2 configurations are previously stored in folder `data`. After processing, the results are stored in other two worksheets in folder `result`, from which data were transcribed to Tables 3 and 4. Respectively:

- The choice of tables are in folder `data`:
  `agr2x2_table3_input.xlsx` and
  `agr2x2_table4_input.xlsx`.
- Results are stored in folder `result`:
  `agr2x2_table3_output.xlsx` and
  `agr2x2_table4_output.xlsx`.

This computation was implemented with `agr2x2_tables_3_4.R`. Some estimators are computed but not transcribed to these tables for reasons explained in the main text. It is the case of the original McNemar's $\chi^2$ [36] and its revisions [31,32], and Hubert's $\mathit{\Gamma}$ that corresponds to $G^2$.

### Creating all 2x2 tables with size $n$

It was implemented `agr2x2_gentablen`, a function to create all possible 2x2 tables of size `n` implemented in `agr2x2_gentablen.R`.

This function returns a list of tables and it is easy to use through a coordinator function that can apply a range of `n` values (`from`...`to`) concatenated in a more convenient data frame and stored in disk as a `csv` file. This procedure is in `agr2x2_createtables.R`.

For instance, to obtain all 2x2 tables with $n = 2$ and $n = 3$:

```
source("agr2x2_createtables.R")

tables <- as.data.frame(
  data.table::fread(agr2x2_createtables(from=2,to=3),header=TRUE)
                       )
```

The content of the dataframe `tables` is:

```
   a b c d n
1  0 0 0 2 2
2  0 0 1 1 2
3  0 0 2 0 2
4  0 1 0 1 2
5  0 1 1 0 2
6  0 2 0 0 2
7  1 0 0 1 2
8  1 0 1 0 2
9  1 1 0 0 2
10 2 0 0 0 2
11 0 0 0 3 3
12 0 0 1 2 3
13 0 0 2 1 3
14 0 0 3 0 3
15 0 1 0 2 3
16 0 1 1 1 3
17 0 1 2 0 3
18 0 2 0 1 3
19 0 2 1 0 3
20 0 3 0 0 3
21 1 0 0 2 3
22 1 0 1 1 3
23 1 0 2 0 3
24 1 1 0 1 3
25 1 1 1 0 3
26 1 2 0 0 3
27 2 0 0 1 3
28 2 0 1 0 3
29 2 1 0 0 3
30 3 0 0 0 3
```

### Computation of Figure 1 and Table 5

The procedure described here was used to compute Figure 1, which applies Holley and Guilford's $G$ as benchmark, complemented by Table 5. However, any other agreement coefficient can be assumed as benchmark to allow comparative studies with the procedures described here.

To create Figure 1 three steps are required: (1) to create all tables with $n = 64$, (2) to compute the inferential decision from all implemented coefficients for each of these tables, and (3) to create figure and table assuming each coefficient as benchmark.

### Creation of all 2x2 tables with $n$=64

We created a file containing all 47,905 possible tables with

```
source("agr2x2_createtables.R")
agr2x2_createtables(from=64,to=64)
```

whose results are stored in `from64to64.csv` (folder `data`, see "Creating all 2x2 tables with size $n$" for 2x2 table creation).

### Inferential tests

Using the functions described in this supplemental material (see "R functions"), inferential decisions were computed and results were stored with a R script

```
source("agr2x2_main.R")
```

To the resulting file, `from64to64.csv` (saved in folder `result`), columns were added to store the computation of all estimators described in the supplemental material (see "R functions").

### Figures and performance checking

It generates a figure with the interval of $\frac{a+d}{n}$ that corresponds to the non-rejection of the null hypothesis for each studied estimator as benchmark. The number of mistakes (discrepancy) with the benchmark is computed and stored in `agr2x2_mistakes_n64.csv` and `agr2x2_report_n64.csv`. The coefficient that minimized the mean `Global` mistakes and has the narrowest interval centered in $\frac{a+d}{n} = 0.5$ was Holley and Guilford's $G$. It corresponds to Figure 1. Table $G$ and Table 5 was extracted from `agr2x2_report_n64.csv`.

The R script for this step is:

```
source("agr2x2_densitygraphs_example.R")
```

**Computation of Table 6**

In order to obtain correlations between estimates, the file `from1to68.csv` stored in folder `result` is required, already containing all estimates computed (see "Computation of Figures 2, 3, 4, and 5").

The procedure `agr2x2_correlations.R` computes Pearson's and Spearman's correlations of Holley and Guilford's $G$ against all other estimators, creating two files in folder `result`:

- `agr2x2_correlations.csv` — all computed correlations.
- `agr2x2_tablehdi.csv` — data summary, transcribed to Table 6.

It also creates files in folder `image`:

- `agr2x2_correlations.pdf` — PDF file containing graphs of correlation values ($r$ and $\rho$) with confidence interval 95% estimated by the R function `cor.test` in function of $1 \leq n \leq 68$.
- multiple scatterplots named agr2x2_rawdata*.png provide maps of behavior analogous to Figure 2, where '*' stands for the agreement coefficient under assessment. These graphs where necessary to verify the stability of each estimator with $1 \leq n \leq 68$.

**Computation of Figures 2, 3, 4, and 5**

The generation of Figures 2, 3, 4, and 5 requires all point-estimates previously computed for all possible tables with $1 \leq n \leq 68$ (a total of 1,028,789 tables), using

```
source("agr2x2_createtables.R")
agr2x2_createtables(from=1,to=68)
```

which must store the resulting file in the folder `data`. Then, we applied the same R script described in this supplemental material, section "Computation of Figure 1 and Table 5", subsection **Inferential tests**", changing the initial parameters (the inferential tests are not required) to:

```
from <- 1
to <- 68
bootstraps <- FALSE
test <- FALSE
```

This procedure adds the necessary columns to store the computation of all estimators (described in "R functions"), saving the results in `from1to68.csv` (folder `result`).

These figures are generated with hexbins applying the packages `ggplot2` and `ggpubr`, a more efficient alternative to plot huge amounts of data than regular scatterplots.

Figure 2 is generated in folder `image` with

```
source("agr2x2_scatterplot_multi.R")
```

The detailed Figures 3, 4, and 5 can also be generated in folder `image` with

```
source("agr2x2_scatterplot_cases.R")
```

This script generates all estimator cases in folder `image`, from which we selected to the main text:

- Figure 3: `agr2x2_scatterplot_case_AC1.pdf`
- Figure 4: `agr2x2_scatterplot_case_k.pdf`
- Figure and 5: `agr2x2_scatterplot_case_MN.pdf`

It is interesting to observe in the figures below that the map of behavior of all estimators do not qualitatively change with increasing number of observations up to $n = 68$, only becoming progressively denser. Therefore, there is no reason to believe that their behavior would change with greater number of observations, nor that we should study tables with greater number of observations. For that reason we elected to detail the behavior of $n = 64$, which is rich in combinations and well above any invocation of the theorem of central limit.

Since it is an important result, for those who do no intend to execute the R script above, this stability of behavior is shown in the following figures:

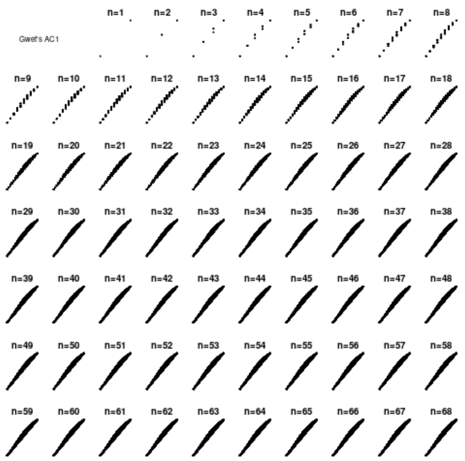

**Fig. 6** Gwet's $AC1$

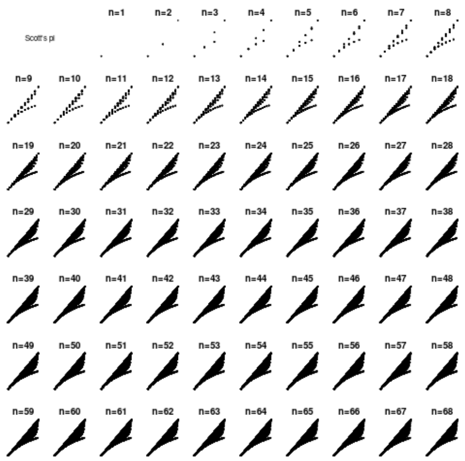

**Fig. 7** Scott's $\pi$

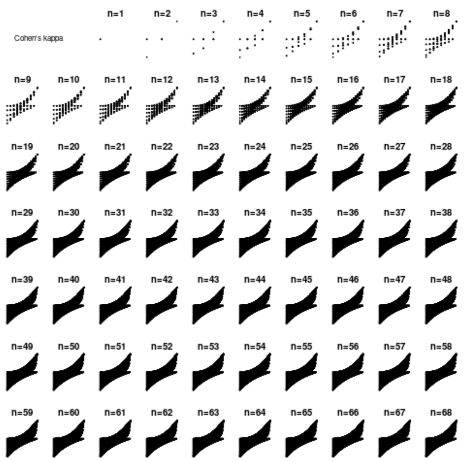

**Fig. 8** Cohen's $\kappa$

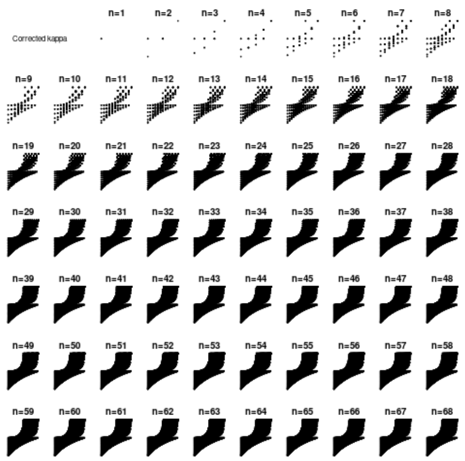

**Fig. 9** Corrected Cohen's $\kappa$ by $\kappa_{max}$

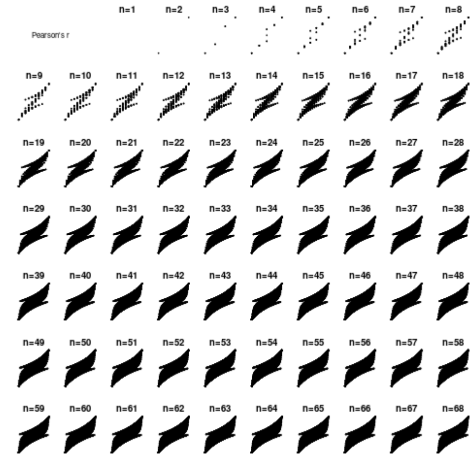


**Fig. 10** Pearson's $r$

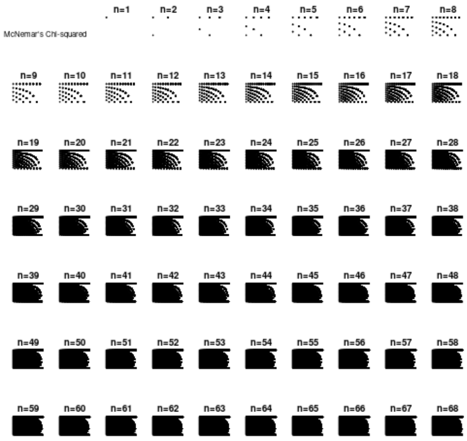


**Fig. 11** McNemar's $\chi^2$

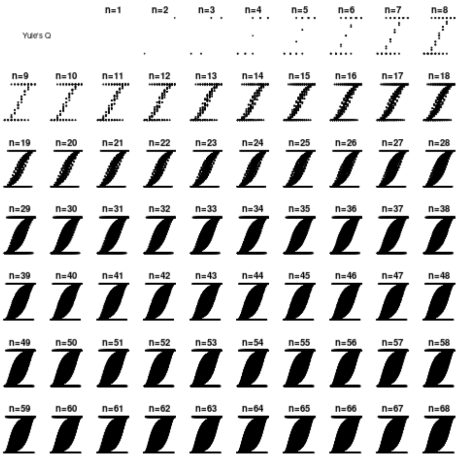


**Fig. 12** Yule's $Q$

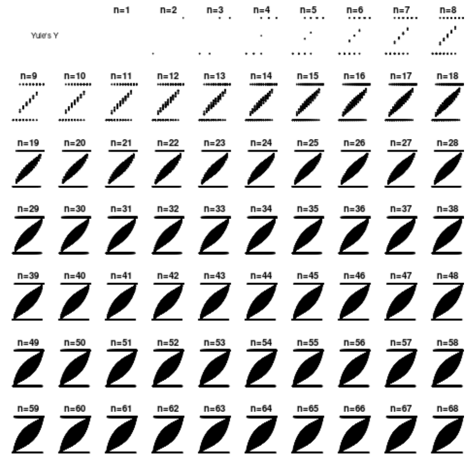


**Fig. 13** Yule's $Y$

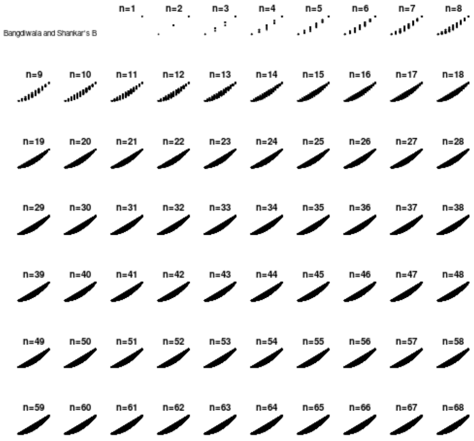


**Fig. 14** Bangdiwala and Shankar's $B$

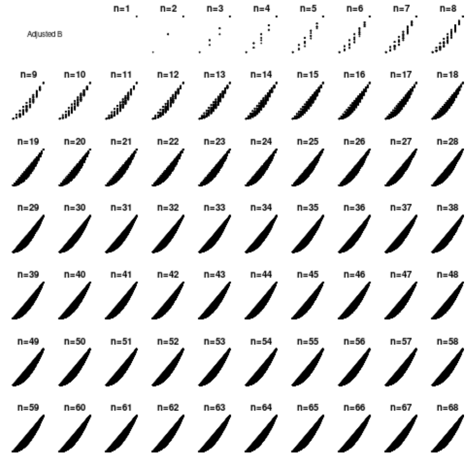


**Fig. 15** Adjusted Bangdiwala and Shankar's $B$

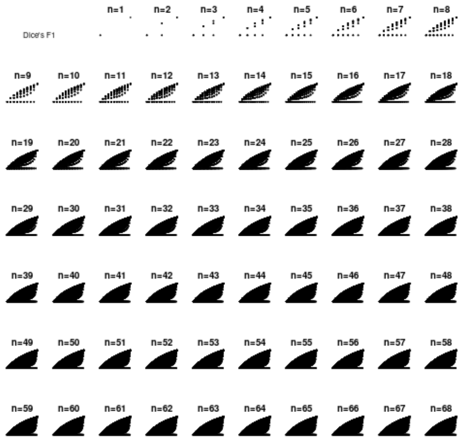


**Fig. 16** Dice's $F1$

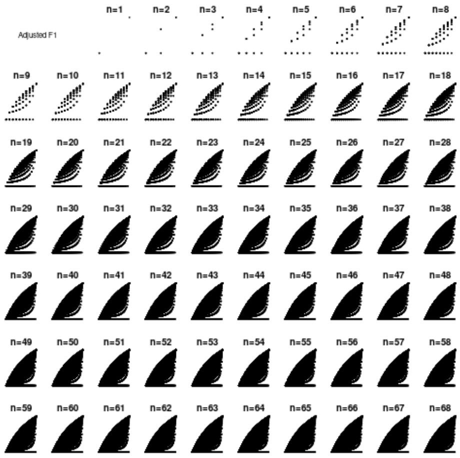


**Fig. 17** Adjusted Dice's $F1$
